# Translational Pharmacokinetic‐Pharmacodynamic Modeling and Efficacious Human Dose Prediction of DNDI‐6148 for the Treatment of Cutaneous Leishmaniasis

**DOI:** 10.1111/cts.70535

**Published:** 2026-04-14

**Authors:** Rasmus Hansen Henninger, Wietse M. Schouten, Byron Arana, Jean‐Yves Gillon, Charles E. Mowbray, Jadel Müller Kratz, Katrien Van Bocxlaer, Thomas P. C. Dorlo

**Affiliations:** ^1^ Department of Pharmacy Uppsala University Uppsala Sweden; ^2^ Department of Pharmacy and Pharmacology Antoni van Leeuwenhoek/the Netherlands Cancer Institute Amsterdam the Netherlands; ^3^ Drugs for Neglected Diseases Initiative Geneva Switzerland; ^4^ Drugs for Neglected Diseases Initiative Latin America Rio de Janeiro Brazil; ^5^ Skin Research Centre, Hull York Medical School, York Biomedical Research Institute University of York York UK

**Keywords:** infectious disease, model based drug development, translational pharmacokinetics‐pharmacodynamics, tropical diseases

## Abstract

Cutaneous leishmaniasis is a neglected tropical disease with only one oral treatment option. DNDI‐6148 is an orally bioavailable compound with potent antiparasitic activity in preclinical studies. Establishing skin target‐site pharmacokinetic/pharmacodynamic (PK/PD) relationships is essential to enable its clinical development. The objective was to characterize the target‐site PK and PD of DNDI‐6148 in a murine *L. major* model and to predict a human efficacious dose. A nonlinear mixed‐effects PK/PD model was developed using data from *L. major* ‐infected BALB/c mice orally administered DNDI‐6148 (6.25–50 mg/kg, bid). The murine PD components and skin distribution characteristics were used jointly with allometrically scaled human PK parameters to predict a clinically efficacious dose. DNDI‐6148 PK in mice was described by a one‐compartment model with dose‐dependent bioavailability, saturable clearance, and a skin‐to‐plasma ratio of 0.56 [95% CI, 0.49–0.68] for both infected and non‐infected skin. Parasite clearance at the infection site followed a sigmoidal *E*_*max*_ relationship, driven by free skin concentrations (*fEC5_0_*: 165 μg/L [95% CI, 125–236]). Human PK parameters for clearance, volume of distribution, and absorption rate were predicted to 3.44 L/h, 79.5 L and 0.360 h^−1^, respectively. DNDI‐6148 doses of 4.0 and 6.0 mg/kg once daily for 14 days were predicted to achieve 95% and 99% parasite reduction from baseline, respectively, in > 90% of simulated patients. This translational PK/PD modeling framework based on a murine infection model of cutaneous leishmaniasis effectively informs human dose selection by accounting for both PK and PD at the skin target site.

## Study Highlights

- What is the current knowledge on the topic?
  ○ Cutaneous leishmaniasis currently has only one approved oral treatment, which is limited by safety and efficacy concerns, and dose selection for novel candidates is complicated by limited understanding of drug exposure at the skin target‐site of infection.
- What question did this study address?
  ○ This study addressed the integration of skin target‐site pharmacokinetic and longitudinal pharmacodynamic data for DNDI‐6148 using a translational model‐informed approach to human dose selection.
- What does this study add to our knowledge?
  ○ The established exposure‐response relationship was integrated with scaled human pharmacokinetics, and a probability of target attainment analysis suggested that daily oral doses of 4.0 or 6.0 mg/kg for 14 days would achieve a high probability of therapeutic success for cutaneous leishmaniasis, reaching parasite reductions of 95% and 99%, respectively.
- How might this change clinical pharmacology or translational science?
  ○ This study presents the first translational modelling framework for cutaneous leishmaniasis that incorporates skin target‐site pharmacokinetics and pharmacodynamics, enabling prediction and exploration of human drug exposure and associated parasite clearance, guiding clinical trial design.

## 1 Introduction

Cutaneous leishmaniasis is the most common manifestation of the neglected tropical disease leishmaniasis, caused by various species of the protozoan parasite genus *Leishmania (L.)*. With an estimated 600,000–1 million new cases annually, cutaneous leishmaniasis disproportionately affects the world's poorest populations [1]. Clinical presentations range from small papules or skin ulcers that may heal spontaneously to chronic skin lesions that can result in disfigurement, psychological distress, and an increased risk of secondary infections. *L. major* , one of more than 20 causative species of cutaneous leishmaniasis, typically causes large, ulcerative lesions with raised borders, which may result in scarring [2]. Miltefosine remains the only oral treatment, yet its prolonged dosing, gastrointestinal adverse effects, and teratogenicity underscore the need for better oral therapies [3].

DNDI‐6148, a benzoxaborole derivative, has emerged as an oral drug candidate for cutaneous leishmaniasis. Its mechanism of action involves inhibition of cleavage and polyadenylation specificity factor 3, an essential endonuclease required for *Leishmania* RNA processing [4]. Preclinical studies have demonstrated its potent in vitro activity against intracellular amastigotes of cutaneous leishmaniasis‐causing *Leishmania* species, with half maximal effective concentration (*EC*_*50*_) values ranging from < 1.1 to 18.3 μM [5]. Preclinically, the compound exhibits moderate *V*_*d*_ (1–2‐fold total body water), a short‐to‐moderate elimination half‐life (3–4 h in rats, 4–7 h in monkeys), hepatic metabolism as the primary *CL* route, and 85%–94% oral bioavailability in both species [4]. Despite this favorable pharmacokinetic (PK) profile, its efficacy in cutaneous leishmaniasis depends on achieving adequate drug exposure at the skin target site of infection, where *Leishmania* parasites reside within macrophages in the dermis. However, there is currently a lack of data on DNDI‐6148's tissue distribution and its ability to achieve target site exposure necessary for parasite clearance. Drug penetration into infected tissues is influenced by many factors, including e.g., protein binding, tissue permeability, and active influx or efflux transport, which can exhibit non‐linear behavior and lead to asymmetry in (unbound) drug concentrations between plasma and tissue. Understanding these dynamics is essential for optimizing dose selection and ensuring effective parasite clearance across leishmaniasis manifestations.

In preclinical models of cutaneous leishmaniasis, pharmacodynamic (PD) markers include lesion size and parasite burden assessed via qPCR or histopathology. These approaches to parasite‐burden assessment require invasive sampling, as collection of target skin tissue necessitates animal sacrifice. In vivo bioluminescence imaging of animals infected with luciferase‐expressing parasites offers a less invasive and more dynamic, longitudinal alternative for evaluating drug candidate efficacy [6].

The objective of this study was to characterize the relationship between plasma and skin target site PK, and subsequently the relationship with skin lesion parasite‐load clearance of DNDI‐6148 in a murine *L. major* infection model, to define the exposure‐response relationship within infected skin tissue and to inform human dose selection based on PK/PD target attainment. Ultimately, this study aims to establish a preclinical PK/PD model that supports the translational development of DNDI‐6148 for cutaneous leishmaniasis treatment.

## 2 Methods

### 2.1 In Vivo Experiments

All animal work was carried out under a UK Home Office project license according to the Animal (Scientific Procedures) Act 1986 and the European Directive 2010/63/EU. The project license (PPL PP1651724) was reviewed by the University of York Animal Welfare and Ethical Review Board prior to submission and consequent approval by the UK. Home Office.

Thirty mice (see Supplementary methods for details) were infected with *L. major* Friedlin:Luc promastigotes. After 14 days, DNDI‐6148 arginine monohydrate was administered via oral gavage at four dosage levels (vehicle control, 6.25, 12.5, 25, and 50 mg/kg, expressed as free base, bid) twice daily for 10 days, with dose intervals varying between approximately 8 and 12 h. Plasma samples were collected on days 1 and 10 at nominal times of 0.5, 1, 2, 4, 8, and 16 h post‐dose (*n* = 3/time point). At the last sampling time, mice were sacrificed and infected/non‐infected skin, liver, and spleen collected (*n* = 6/group/tissue). Concentrations in plasma and tissue were quantified using a validated UPLC–MS/MS assay [7]. Bioluminescence imaging of skin was performed using an IVIS Spectrum Imaging System (Revvity) on days 1–4, 6, 8, and 10, and lesion size was measured daily using digital calipers.

### 2.2 PK/PD Model Development

NONMEM (version 7.5, ICON Development Solutions, Ellicott City, MD, USA) and Perl‐speaks‐NONMEM (PsN, version 5.4.0) were used for nonlinear mixed‐effects modeling, with Pirana (version 21.11.1) as the interface [8, 9]. First‐order conditional estimation with interaction was used for parameter estimation. Data processing was performed using R (version 4.4.1). Model development was performed in four consecutive steps using the PPP&D approach: (1) plasma PK, (2) tissue PK, (3) exposure‐parasite relationship in skin, and (4) parasite clearance‐lesion size relationship [10]. Below‐limit‐of‐quantification (BLQ) data were handled using the M6 method [11, 12]. Between‐subject variability (BSV) was evaluated on all parameters, implemented using an exponential model.

Model selection was guided by physiological plausibility, difference in the objective function value (dOFV), parameter precision, goodness‐of‐fit (GOF) plots, and simulation‐based (*n* = 1000) visual predictive checks (VPCs) [13, 14, 15]. The likelihood ratio test was used to assess the statistical significance of including additional parameters in nested models, assuming the objective function value (OFV) follows a *χ* ^2^ distribution. A dOFV of ≥ 3.84 was considered statistically significant at the 5% level (*p* < 0.05, with 1 degree of freedom).

#### 2.2.1 Plasma Pharmacokinetics

One and two‐compartment models with first‐order absorption and linear or saturable elimination were evaluated to describe murine plasma pharmacokinetics of DNDI‐6148. The effect of body weight on *CL* and *V*_*d*_ was incorporated using allometric scaling, as shown in Equations 1 and 2; [16]:

(1) $$CL_{i}=CL_{\mathrm{pop}}\cdot {\left(\frac{\mathrm{WT}}{22}\right)}^{0.75}$$

(2) $${V_{d}}_{i}={V_{D}}_{\mathrm{pop}}\cdot {\left(\frac{\mathrm{WT}}{22}\right)}^{1.0}$$

where $CL_{i}$ and ${V_{d}}_{i}$ are the individual parameters for each mouse, and *WT* (g) denotes the murine body weight. $CL_{\mathrm{pop}}$ and ${V_{d}}_{\mathrm{pop}}$ are the typical parameters for a mouse normalized to the median body weight (i.e., 22 g). As only oral data were available, *CL* and *V*_*d*_ were estimated relative to oral bioavailability (*F*).

#### 2.2.2 Tissue Distribution

Tissue penetration of DNDI‐6148 was described using separate effect compartments, assuming no mass transfer from the plasma compartment, with the following Equation 3; [17]:

(3) $$\frac{dC_{\text{tissue}}}{\mathrm{dt}}=k_{\text{plasma}-\text{tissue}}\cdot \left(R_{\text{tissue}-\text{plasma}}\cdot \frac{A_{\text{plasma}}}{V_{\text{plasma}}}-C_{\text{tissue}}\right)$$

in which *C*_*tissue*_ denotes the drug concentration (ng/g tissue) within tissue (infected/non‐infected skin, liver or spleen), *k*_*plasma‐tissue*_ is an intercompartmental rate constant for transfer of drug from plasma to tissue, *R*_*tissue‐plasma*_ is the penetration coefficient between tissue and plasma, and *A*_*plasma*_ */V*_*plasma*_ represents drug concentration in plasma at time *t*. *k*_*plasma‐tissue*_ was fixed to 20 h^−1^, assuming a near‐instantaneous drug penetration as tissue concentrations were only available from a single time point. Disease state (infected/non‐infected) was evaluated as a categorical covariate on *R*_*skin‐plasma*_ to determine whether drug penetration differed between infected and non‐infected skin.

#### 2.2.3 Drug‐Induced Parasite Elimination and Lesion Healing Dynamics

No pharmacodynamic data were available before treatment. To describe natural parasite growth and death, a turnover model or baseline parasite load estimation was considered using control data (*n* = 6). The unbound concentrations of DNDI‐6148 at the target skin site over time, as predicted by the PK model, were assumed to drive drug‐induced parasite clearance. The fraction unbound (*fu*) in plasma for BALB/c mice and humans has been determined to be 6.6% (in‐house data), and a similar protein binding for skin tissue was assumed.

The drug effect was implemented as a sigmoidal maximum effect (*E*_*max*_) model, hypothesized to enhance the parasitic killing rate ($k_{\text{kill}}$), using Equations 4 and 5:

(4) $$k_{\text{kill}}=\frac{E_{\max}\cdot C_{\text{skin}}^{\gamma }\left(t\right)\cdot \mathrm{fu}}{{\mathrm{EC}}_{50}^{\gamma }+C_{\text{skin}}^{\gamma }\left(t\right)\cdot \mathrm{fu}}$$

(5) $$\frac{dA_{\text{parasite}}}{\mathrm{dt}}=-k_{\text{kill}}\cdot A_{\text{parasite}}$$

where $E_{\max}$ is the maximum drug effect on parasite killing, $C_{\text{skin}}^{\gamma }\left(t\right)$ is the total or unbound drug concentration in the skin at time t, ${\mathrm{EC}}_{50}^{\gamma }$ is the concentration of total or unbound drug (${\mathrm{fEC}}_{50}^{\gamma }$) in the skin at which 50% of the maximum effect is achieved, γ is the Hill coefficient, and $A_{\text{parasite}}$ denotes the parasite bioluminescence at a given timepoint. A delay between drug exposure and parasite clearance was evaluated.

A relationship between lesion size and parasite burden was assumed, with larger lesions corresponding to higher parasite loads. Direct, delayed sigmoidal *E*_*max*_, and linear models were evaluated to describe the relationship, such as the linear model shown in Equation 6:

(6) $$\frac{dA_{\text{lesion}}}{\mathrm{dt}}=\text{slope}\cdot A_{\text{parasite}}\cdot A_{\text{lesion}}-k_{\text{heal}}\cdot A_{\text{lesion}}$$

where slope is the linear constant, $A_{\text{lesion}}$ is the lesion size at a given timepoint and $k_{\text{heal}}$ is the first‐order lesion healing rate.

#### 2.2.4 Exposure and Target Attainment

Secondary PK parameters were derived from individual Empirical Bayes estimates (EBEs) from the final murine PK/PD model by simulating individual concentration–time profiles using the post hoc parameter estimates and integrating these profiles over the relevant interval. The AUC from treatment start to day 10 in plasma and skin was calculated for both free (*fAUC_0–D10_*) and total concentrations (*AUC_0–D10_*), as was the percentage of time that free concentrations remained above the model‐estimated *fEC*_*50*_ in infected skin; steady‐state AUC based on total plasma concentrations (*AUC_ss_*_,_ *_0–24h_*) was also determined for comparison with toxicology studies in other species.

#### 2.2.5 Prediction of Human PK

The typical PK parameters (*k_a_*, *CL/F*, and *V*_*d*_ */F*) estimated from mice were extrapolated to humans using a single‐species allometric approach, with fixed exponents of 0.75, 1.00, and −0.25 for *CL/F*, *V*_*d*_ */F*, and *k_a_*, respectively [18, 19]. To account for interspecies differences in protein binding, *CL* and *V*_*d*_ were corrected for *fu* in each species [20]. Species‐specific PK differences were evaluated for their physiological relevance in humans; saturable clearance and dose‐dependent bioavailability were considered mouse‐specific and were therefore excluded from the extrapolated model.

#### 2.2.6 Prediction of Human Efficacy

Predicted human PK parameters were combined with tissue distribution characteristics and PD components from the murine PK/PD model and used to simulate the antiparasitic effect of DNDI‐6148 in humans. Unbound skin concentrations were used to drive the antiparasitic effect. An *fu* of 0.07 in plasma was applied, and a similar *fu* in tissue was assumed. Initial parasite loads in humans were set to the same as in mice. PTA for 95% and 99% parasite reduction from baseline in humans was determined by simulating 10,000 human PK/PD profiles per dose scenario, incorporating BSV: 37% coefficient of variation (*CV*) on *R_skin–plasma_*, 60% *CV* on parasite load at baseline, and an inflated 30% *CV* on *CL*. Residual variability was excluded. Simulations were performed for 70 kg adults receiving once‐daily doses of 3–20 mg/kg for 7–14 days (1.0 mg/kg increments) or twice‐daily doses of 1.5–7.0 mg/kg for 7–10 days (0.5 mg/kg increments).

## 3 Results

### 3.1 Data

**FIGURE 1:**
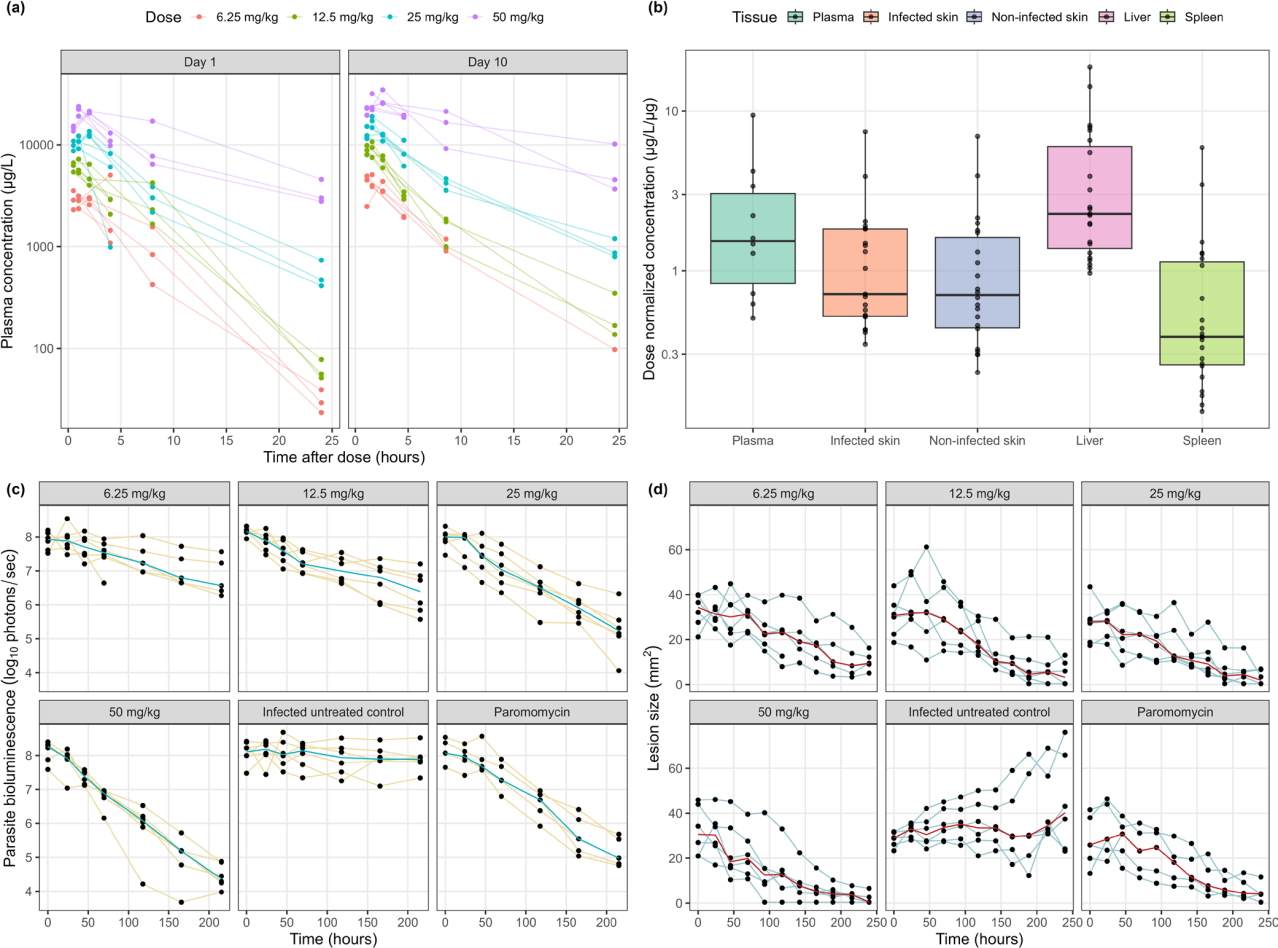
Plasma, tissue, parasite bioluminescence and lesion size observations. (a) Total plasma concentration‐time profiles following administration of different dose levels of DNDI‐6148 (6.25 mg/kg (red), 12.5 mg/kg (green), 25 mg/kg (blue), and 50 mg/kg (purple)) on Day 1 (left) and Day 10 (right). (b) Dose‐normalized concentration (μg/L/μg) of DNDI‐6148 across different tissues: Plasma (teal), infected skin (orange), non‐infected skin (blue), liver (pink), and spleen (green). Box plots show the median (horizontal line), interquartile range (box), and whiskers extending to 1.5 × interquartile range. Black dots represent individual data points, with those beyond the whiskers indicating outliers. The y‐axis is on a logarithmic scale. (c) Observed parasite bioluminescence reduction over a 10‐day period in the infected untreated control, paromomycin (50 mg/kg, qd, IP), and different dose levels of DNDI‐6148 (6.25 mg/kg, 12.5 mg/kg, 25 mg/kg, and 50 mg/kg; bid, PO). Data points and yellow lines represent individual observations, while the blue line indicates the median parasite bioluminescence. (d) Lesion size progression over a 10‐day period in the infected untreated control, paromomycin (50 mg/kg, qd, IP), and different dose levels of DNDI‐6148 (6.25 mg/kg, 12.5 mg/kg, 25 mg/kg, and 50 mg/kg; bid, PO). Lesion size is presented as calculated areas (mm^2^). Data points and blue lines represent individual observations, while red lines indicate the median lesion size for each group.

A total of 141 plasma, 43 skin (21 infected, 22 non‐infected), 23 liver, and 23 spleen PK samples, along with 207 parasite bioluminescence and 325 lesion‐size PD measurements, were collected from 30 mice (24 treated, 6 controls). No plasma PK samples or parasite bioluminescence data were BLQ. Three skin tissue PK samples (7.0%) and 22 lesion‐size measurements (8.5%) were BLQ. An overview of the data included in this analysis can be seen in Figure 1a,b.

### 3.2 Population PK/PD Model

#### 3.2.1 Plasma Pharmacokinetics

**FIGURE 2:**
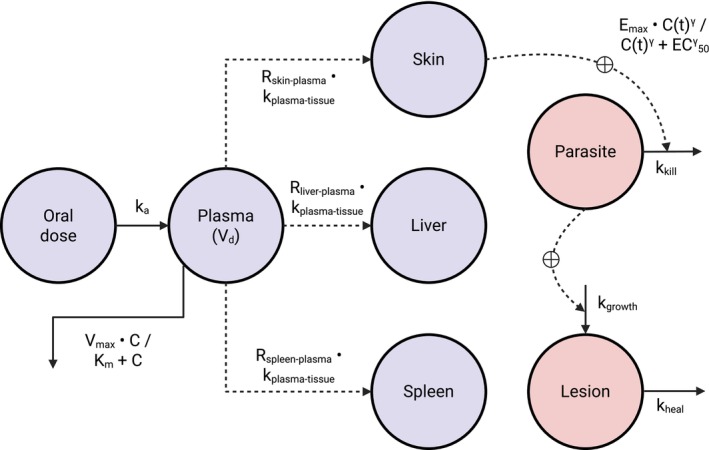
Structural outline of the PK/PD model. C, concentration; *EC*_*50*_, half‐maximal effect concentration; *E*_*max*_, maximum effect; *k*_*a*_, first‐order absorption rate; *k*_*growth*_, lesion growth rate; *k*_*heal*_, lesion healing rate constant; *k*_*kill*_, parasite kill rate; *k*_*m*_, Michaelis–Menten constant; *k*_*plasma‐tissue*_, plasma‐to‐tissue distribution rate; *R*_*liver:Plasma*_, liver‐to‐plasma ratio; *R*_*skin:Plasma*_, skin‐to‐plasma ratio; *R*_*spleen:Plasma*_, spleen‐to‐plasma ratio; *V*_*d*_, volume of distribution; *V*_*max*_, maximum elimination rate; *γ*, Hill coefficient.

The plasma PK of DNDI‐6148 in mice was best described by a one‐compartment disposition model with first‐order absorption. A dose‐dependent decrease in relative bioavailability, estimated using a power function, was significant (dOFV = −35.4, df = 1), resulting in a less‐than‐proportional increase in exposure with increasing dose. Compared to the 6.25 mg/kg dose level, relative bioavailability, decreased to 79.1%, 62.6%, and 49.5% at 12.5, 25, and 50 mg/kg, respectively. Elimination was best described by concentration‐dependent, saturable Michaelis–Menten kinetics (dOFV = −45.4, df = 1), with a *V*_*max*_/*F* of 65.4 μg/h [95% CI, 55–77] and an apparent Michaelis–Menten constant *K*_*m*_ of 8000 μg/L [95% CI, 6500–9500]. Final population PK parameter estimates are summarized in Table 1, with a structural outline shown in Figure 2.

**TABLE 1:** Population parameter estimates of the final murine PK model.

Parameter	Description	Unit	Estimate	95% CI^a^
Fixed effect parameters				
*k*_*a*_	Oral absorption rate constant	h^−1^	2.7	2.0–3.9
*V*_*max*_/*F* ^b^	Apparent maximal elimination rate	μg/h	65	55–77
*K*_*m*_	Apparent Michaelis–Menten constant	μg/L	8.0 × 10^3^	6.5 × 10^3^–9.5 × 10^3^
*V*/*F* ^b^	Apparent volume of distribution	L	0.025	0.022–0.028
*F*_*rel*_	Relative bioavailability		1.0	Fixed
*θ*_*dose*_ ^c^	Power dose effect on relative bioavailability		−0.34	−0.39 – −0.28
*k*_*plasma‐tissue*_	Transfer rate of drug from plasma to tissue	h^−1^	20	Fixed
*R*_*skin‐plasma*_	Skin to plasma penetration coefficient		0.56	0.49–0.68
*R*_*liver‐plasma*_	Liver to plasma penetration coefficient		1.9	1.6–2.2
*R*_*spleen‐plasma*_	Spleen to plasma penetration coefficient		0.34	0.28–0.41
Between‐subject variability (BSV)				
*K*_*m*_	BSV on Michaelis–Menten constant	*CV*%	6.5	3.7–8.6
*R*_*skin‐plasma*_	BSV on skin to plasma penetration coefficient	*CV*%	37	23–47
*R*_*liver‐plasma*_	BSV on liver to plasma penetration coefficient	*CV*%	30	21–42
*R*_*spleen‐plasma*_	BSV on spleen to plasma penetration coefficient	*CV*%	44	34–67
Residual unexplained variability (RUV)				
*add*_*plasma*_	Additive residual error for plasma	μg/L	2.5	Fixed
*prop*_*plasma*_	Proportional residual error plasma	*CV*%	36	31–42

Abbreviations: CI, confidence interval; *CV*, coefficient of variation, approximated using $\sqrt{\exp^{\text{OMEGA}}-1}$.

^a^ 95% confidence intervals obtained using Sampling Importance Resampling (SIR) algorithm as implemented in Perl‐speaks‐NONMEM.

^b^ Allometrically scaled based on body weight with power exponent of 0.75 for clearance and 1 for volume of distribution. Estimate is given for a standardized weight of 22 g.

^c^ Dose‐dependency in bioavailability was parameterized by scaling the bioavailability relative to the 6.25 mg/kg dose, $F={\left(\frac{\text{dose}}{6.25\frac{\mathrm{mg}}{\mathrm{kg}}}\right)}^{\theta_{\text{dose}}}$.

#### 3.2.2 Tissue Distribution

Skin distribution was described by a separate compartment, with the *R_skin–plasma_* estimated at 0.56 [0.49, 0.68] and a moderate variability of 37% *CV*, indicating reduced penetration from plasma to skin. There was no difference between infected and non‐infected skin in terms of total drug skin distribution. Tissue distribution into the liver and spleen was estimated at 1.91 [1.6–2.2] and 0.34 [0.28–0.41], respectively, indicating significant drug accumulation in the liver and reduced penetration into the spleen compared to plasma (see Table 1 for a summary of tissue distribution).

#### 3.2.3 Drug‐Induced Parasite Elimination, and Lesion Healing Dynamics

Parasite bioluminescence correlated well with qPCR‐derived parasite load in lesion tissue (Pearson's *R* = 0.94; Figure S1), performing better than lesion size (*R* = 0.65; Figure S2) at the end of treatment. qPCR scaled proportionally with the bioluminescence signal (log_10_[qPCR/mg] = −1.00 + 0.83 log_10_[BLI]; RSD 0.39, *R* ^2^ = 0.89); where a bioluminescence of 8 log_10_ photons/s corresponded to 4.2 × 10^5^ parasites per mg of skin.

Parasite proliferation was best described by a constant baseline, estimated at 8.01 log_10_ photons/s [7.9–8.1] with an associated high BSV of 60% *CV*, assuming no net growth and suggesting the parasite burden had reached a steady state at the start of treatment.

The skin parasite loads in the different treatment groups all demonstrated a significant reduction over time compared to the vehicle control. Drug‐induced parasite elimination was best characterized by a sigmoidal *E*_*max*_ model, with no delay, directly linked to the free skin target site concentration over time (Equation 4), with the effect being proportional to the drug concentration. The *E*_*max*_ was estimated at 0.049 h^−1^ [0.042–0.058], with a half‐maximal effect achieved at *fEC*_*50*_ of 165 μg/L [125–236]. Figure S3 shows simulated unbound skin concentration–time profiles (6.25–50 mg/kg, bid for 10 days) relative to the estimated in vivo *L. major* *fEC_50_*.

The lesion size–parasite burden relationship was best described by modeling the lesion growth rate as proportional to the parasite burden, as shown in Equation 6, with a slope estimated at 0.0029 ± 2.78 × 10^−4^/photons [0.0010–0.0050]. The lesion healing rate was estimated at 0.0272 h^−1^. The baseline lesion size was estimated at 31 mm^2^, with a moderate BSV of 34.2% *CV*. Final PD parameter estimates are summarized in Table 2.

**TABLE 2:** Population parameter estimates of the final murine PD model.

Parameter	Description	Unit	Estimate	95% CI^a^
Fixed effect parameters				
*Parasite*_*bas*e_	Baseline skin parasite bioluminescence	log_10_ photons/s	8.0	7.9–8.1
*E*_*max*_	Maximal rate of parasite elimination	h^−1^	0.049	0.042–0.058
*fEC*_*50*_	Free drug concentration in skin needed to achieve 50% of *E*_*max*_	μg/L	165	125–236
γ	Sigmoidicity factor		1.6	1.1–2.8
*Lesion*_*base*_	Baseline lesion size	mm^2^	31	26–36
*slope*	Parasite‐induced lesion growth slope factor	2.78 × 10^−4^/photons	0.0029	0.0010–0.0050
*k*_*heal*_	Lesion healing rate	h^−1^	0.027	0.010–0.044
Between‐subject variability (BSV)				
*Parasite*_*base*_	BSV on baseline skin parasite bioluminescence	*CV*%	60	45–94
*Lesion*_*base*_	BSV on baseline lesion size	*CV*%	35	26–43
Residual unexplained variability (RUV)				
*log*_*10*_ *add*_*parasite*_	Additive residual error for parasite bioluminescence	log_10_ photons/s	0.13	0.045–0.075
*add*_*lesion*_	Additive residual error for lesion size	mm^2^	0.39	Fixed
*prop*_*lesion*_	Proportional residual error for lesion size	*CV*%	37	30–43

Abbreviations: CI, confidence interval; *CV*, coefficient of variation, approximated using $\sqrt{\exp^{\text{OMEGA}}-1}$.

^a^ 95% confidence intervals obtained using Sampling Importance Resampling (SIR) algorithm as implemented in Perl‐speaks‐NONMEM.

#### 3.2.4 Model

VPCs (Figures S4–S6) and GOF plots (Figures S7–S10) for the plasma, skin, parasite, and lesion models showed no major misspecification and indicated adequate predictive performance across dose groups. Parameter estimate uncertainty was generally low for all parameters (%RSE < 30%, narrow CIs) except for lesion‐related parameters, which showed wider CIs and higher relative standard errors (slope: 42%, $k_{\text{heal}}$: 32%).

#### 3.2.5 Exposure and Target Attainment

Exposure and target attainment in plasma and infected skin, based on individual EBEs, are summarized in Table 3. Simulated parasite load reductions are shown in Figure S11. On average, 50 mg/kg achieved 95% and 99% parasite reduction by days 3 and 4, respectively. At 25 mg/kg, these targets were reached by days 4 and 6, while 95% reduction was achieved by day 7 at 12.5 mg/kg. PD targets were not achieved at 6.25 mg/kg.

**TABLE 3:** Plasma and infected skin exposure and target attainment of DNDI‐6148 across dose levels.

Parameter	6.25 mg/kg	12.5 mg/kg	25 mg/kg	50 mg/kg
Plasma exposure				
*fAUC*_*0–D10*_ (h μg/mL)	29.80 (27.25–32.01)	56.54 (51.75–64.04)	117.7 (104.8–123.1)	291.6 (275.3–311.4)
*AUC*_*0–D10* (h μg/mL)_	451.5 (412.9–485.0)	856.7 (784.1–970.3)	1783 (1588–1865)	4419 (4171–4719)
*AUC*_*ss,0–24h* (h μg/mL)_	45.23 (41.36–48.58)	85.82 (78.55–97.20)	178.6 (159.0–186.8)	442.6 (417.8–472.7)
Infected skin exposure and target attainment				
*fAUC*_*0–D10*_ (h μg/mL)	22.86 (14.87–31.10)	35.92 (23.62–44.57)	69.56 (45.00–76.68)	121.6 (99.43–295.6)
*AUC*_*0–D10*_ (h μg/mL)	346.4 (225.4–471.2)	544.2 (357.9–675.4)	1054 (681.9–1162)	1843 (1506–4478)
Time > *fEC*_*50*_ (%)	22.72 (0.3994–34.30)	40.34 (20.63–48.36)	71.90 (54.18–77.34)	98.21 (96.24–99.96)

*Note:* Values are presented as median (range).

Abbreviations: *fAUC*_*0‐D10*_, free area under the concentration‐time curve from treatment start until day 10 (last day of dosing); *AUC*_*0‐D10*_, area under the total concentration‐time curve from treatment start until day 10 (last day of dosing); *AUC*_*ss,0–24h*_, area under the total plasma concentration‐time curve from treatment start to 24 h at steady state; Time > *fEC*_*50*_, percentage of the total time that DNDI‐6148 concentration remained above the model‐estimated free susceptibility value *fEC*_*50*_ (165 μg/L) in infected skin.

Simulated lesion‐size reduction (Figure S12) showed all dose levels were predicted to significantly reduce lesion size during the 10 days of treatment, with higher dose levels achieving more rapid parasite elimination and, consequently, faster lesion healing with less variability. Simulated median lesion‐size reduction versus control at end of treatment was 74% [95% CI, 56–85], 81% [68–90], 89% [79–94], and 94% [90–96] for 6.25, 12.5, 25, and 50 mg/kg, respectively. A 10‐day bid regimen of 17.5 mg/kg and 25 mg/kg was predicted to be sufficient to achieve 95% and 99% parasite reduction in more than 95% of mice, respectively.

#### 3.2.6 Prediction of Human PK

The *k*_*a*_, total *CL*, and total *V*_*d*_ values predicted by allometric scaling for a 70‐kg human were 0.360 h^−1^, 3.44 L/h, and 79.5 L, respectively. Simulated human exposure for 4.0 and 6.0 mg/kg qd over 14 days resulted in median *AUC*_*ss,0–24h*_ values of 81.6 and 122 h μg/mL and cumulative *AUC*_*0–14d*_ values of 1091 and 1632 h μg/mL, respectively, based on total plasma concentrations.

#### 3.2.7 Prediction of Human Efficacy

**FIGURE 3:**
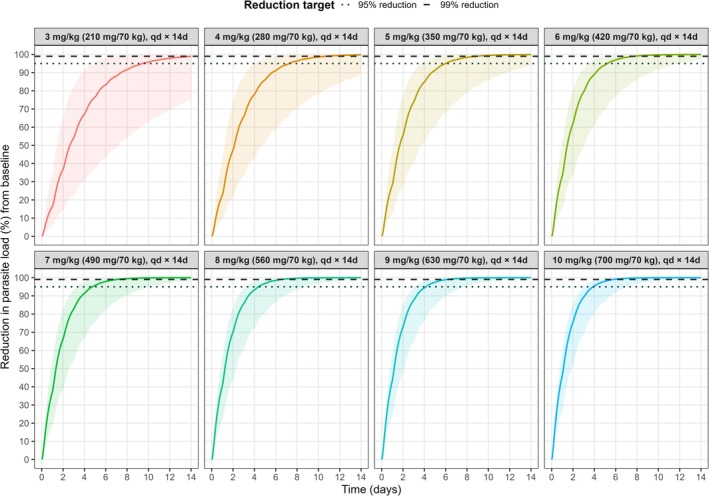
Model‐predicted *L. major* parasite load reduction in humans following 14 days of qd DNDI‐6148 treatment at doses ranging from 3.0 to 10 mg/kg (*n* = 10,000 per dose scenario), based on the final human PK/PD model. The bold line represents the median; the shaded area indicates the 95% confidence interval. Qd, once daily.

**FIGURE 4:**
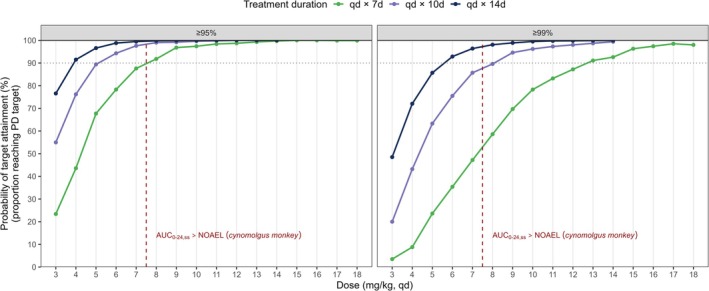
Model‐predicted PTA for *L. major* parasite load reduction in humans following qd DNDI‐6148 treatment for 7 (green), 10 (purple), or 14 (blue) days at doses ranging from 3 to 18 mg/kg (*n* = 10,000 per dose). The horizontal dashed line indicates that 90% of the simulated population has reached the specified target (95% or 99% parasite reduction from baseline), and the vertical dashed line represents the dose at which the model‐predicted human *AUC*_*0–24h, ss*_ exceeds that of the preclinical NOAEL. PTA, probability of target attainment; d, days; NOAEL, no‐observed adverse effect level; Qd, once daily.

Dose regimens of 4.0 and 6.0 mg/kg qd DNDI‐6148 for 14 days were predicted to be sufficient to achieve 95% and 99% parasite reduction from baseline in > 90% of simulated patients, respectively. In line with the target product profile for cutaneous leishmaniasis as a 7‐day oral regimen, 8.0 and 13 mg/kg qd for 7 days were predicted to achieve similar efficacy. With bid dosing, 4.0 mg/kg for 7 days achieved > 90% PTA for 95% parasite reduction, while 4.0 mg/kg for 10 days achieved > 90% PTA for 99% reduction. The predicted reductions in parasite load from baseline are shown in Figure 3. Predicted PTA across dosing scenarios, treatment durations, and reduction targets for qd treatment are shown in Figure 4, with corresponding bid results in Figure S13. All PTA outcomes are summarized in Table S1.

## 4 Discussion

In this study, we characterized the plasma and tissue PK of DNDI‐6148 and evaluated its target‐site exposure–response relationships in an *L. major* ‐infected cutaneous leishmaniasis mice model using a nonlinear mixed‐effects modeling approach. The established exposure‐response relationship was integrated with allometrically scaled human PK, and PTA analysis suggested that clinical dosing regimens of 4.0 or 6.0 mg/kg qd for 14 days would achieve a high probability of therapeutic success, for parasite reduction targets of 95% and 99%, respectively. Under DNDi's target product profile of a 7‐day oral regimen, doses of 8.0 and 13 mg/kg qd were predicted to achieve parasite clearance. Parasite reduction targets were selected per EMA antimicrobial guidance to support flexible dose selection, and simulations indicated that both targets were compatible with significant lesion resolution [21]. In cutaneous leishmaniasis, sterilizing cure is typically not considered feasible, as parasites often persist despite lesion healing [22].

We identified several non‐linearities in the plasma pharmacokinetics of DNDI‐6148 in mice. The estimated *CL* varies with drug concentration; however, at lower concentrations, *CL* in mice (6.19 mL/min/kg) is comparable to SD rats (4.5 mL/min/kg) and beagle dogs (13.7 mL/min/kg). Mouse *V*_*d*_ (1.1 L/kg) was about twice that of rats (0.6 L/kg) and slightly lower than dogs (1.3 L/kg) (3). The dose‐dependent decrease in bioavailability at higher doses likely reflects limited solubility under physiologically relevant conditions. Similar non‐linearities in mice and hamsters have been described elsewhere [23]. In a recent Phase 1 study of DNDI‐6148 up to 380 mg (equivalent to 5.4 mg/kg for a 70‐kg human), plasma AUC and *C*_*max*_ increased less than proportionally with dose, while *CL* saturation was not evident [24].

This study provides the first in vivo data on the distribution of DNDI‐6148 into the major target organs of *Leishmania* infection. Notably for cutaneous leishmaniasis, skin exposure was around 61% of plasma exposure in terms of AUC, with no significant difference in total drug concentration between infected and non‐infected skin. Our results suggest that drug concentrations in non‐infected skin tissue may serve as a suitable surrogate for determining exposure–response relationships at the infected skin. In the liver and spleen, key target organs for visceral leishmaniasis, drug accumulation was substantial in the liver but lower in the spleen relative to plasma. A study limitation is that *k*_*plasma–tissue*_ could not be estimated, as tissues were obtained from a single time point, precluding assessment of distribution delays; however, AUCs are generally less affected by such delays than *C*_*max*_ [17].

The exposure‐response relationship was well described with a direct sigmoidal *E*_*max*_ model, and bioluminescence as a quantitative parasite‐burden biomarker was validated against paired qPCR. The model‐estimated *fEC*_*50*_ (165 μg/L), falls within the *fEC*_*50*_ values (range: 36–1089 μg/L) obtained from various intracellular macrophage amastigote *L. major* in vitro assays [5]. Lesion growth rate was modeled as proportional to parasite burden with a constant healing rate (*k*_*heal*_). *k*_*heal*_, estimated at 0.027 h^−1^ [0.010–0.044], corresponds to a typical skin turnover time (1/k) of ~1.5 days, faster than epidermal turnover in non‐diseased BALB/c mice (8–10 days), suggesting inflammation‐driven increases in cellular turnover and tissue remodeling [25].

Human PK was predicted using single‐species allometric scaling from mouse. Since saturation in *CL* was observed only in mice, Michaelis–Menten–type elimination was omitted, and *CL* for the purpose of allometric scaling was estimated as *V*_*max*_/*F*/*K*_*m*_. The predicted *CL*/*F* of 3.44 L/h was approximately 1.05‐ to 1.52‐fold higher than the mean human *CL*/*F* determined by non‐compartmental analysis on the recently concluded Phase 1 trial (range: 2.26–3.28 L/h), while the predicted *V*_*d*_/*F* of 79.5 L was within 0.76‐ to 1.49‐fold of the observed human range (53.2–104 L). The human plasma *AUC*_*0–24h*_ based on the extrapolated human PK parameters from this study for doses tested in this Phase 1 trial (10–380 mg) were within a 0.66‐ to 1.15‐fold range of the geometric mean of the reported Phase 1 *AUC*_*0–24h*_ [24]. Human PK parameters were thus reasonably predicted, with all estimates falling within or near the 0.5–2‐fold range.

Toxicity studies in the most sensitive species, cynomolgus monkeys, established a no‐observed‐adverse‐effect level (NOAEL) of 15 mg/kg/day (*AUC*_*ss,0–24h*_: 162 and 173 h μg/mL in males and females, respectively). Simulated median human *AUC*_*ss,0–24h*_ values at proposed doses of 4.0 and 6.0 mg/kg were below NOAEL exposures in all preclinical species. In a Phase 1 study, single oral doses up to 380 mg (5.4 mg/kg) were well tolerated in healthy male subjects (*n* = 8), with no serious adverse events [24]. The 4.0 and 6.0 mg/kg doses are within or slightly above the highest Phase 1 dose.

This is the first translational PK/PD modeling framework for cutaneous leishmaniasis based on murine *L. major* infection data that incorporates target site PK and PD, and can be used to predict and explore scenarios of drug exposure and parasite clearance in humans, with the potential of guiding Phase II clinical trial design and showcasing the power of model‐informed drug development.

## Author Contributions

All authors wrote the manuscript; B.A., J.‐Y.G., C.E.M., J.M.K., K.V.B., and T.P.C.D. designed the research; K.V.B., W.M.S. and R.H.H. performed the research; R.H.H. and T.P.C.D. analysed the data.

## Funding

The project activities were supported by Dioraphte Foundation (Oegstgeest, Netherlands). DNDi is grateful to its donors, public and private, who have provided funding for all DNDi activities since its inception in 2003. A full list of DNDi's donors can be found at http://www.dndi.org/about/donors/. TD was supported by the Swedish Research Council (VR grant number 2022–01251). KVB was supported by a fellowship awarded from the Research Council United Kingdom Grand Challenges Research Funder under grant agreement A Global Network for Neglected Tropical Diseases grant number MR/P027989/1.

## Conflicts of Interest

The authors declare no conflicts of interest.

## Supporting information


**Figure S1:** Correlation between parasite load by qPCR and bioluminescence.
**Figure S2:** Correlation between parasite load by qPCR and lesion size.
**Figure S3:** Simulated concentration‐time profiles.
**Figure S4:** Dose‐stratified visual predictive checks.
**Figure S5:** VPC parasite bioluminescence.
**Figure S6:** VPC lesion size reduction.
**Figure S7:** Goodness‐of‐fit plasma PK model.
**Figure S8:** Goodness‐of‐fit tissue‐to‐plasma ratios.
**Figure S9:** Goodness‐of‐fit parasite bioluminescence.
**Figure S10:** Goodness‐of‐fit lesion size model.
**Figure S11:** Model‐predicted parasite reduction.
**Figure S12:** Model‐predicted lesion size reduction.
**Figure S13:** Probability of target attainment for *L. major* parasite load reduction in humans following bid treatment for 7 or 10 days.
**Table S1:** PTA results.
