# Supplementary material for: Translational Pharmacokinetic‐Pharmacodynamic Modeling and Efficacious Human Dose Prediction of DNDI‐6148 for the Treatment of Cutaneous Leishmaniasis
Source: Clin Transl Sci. 2026 Apr 14;19(4):e70535. doi: 10.1111/cts.70535 (PMC13077782; doi:10.1111/cts.70535)
Supplement: Supplementary file 1 — Figure S1: Correlation between parasite load by qPCR and bioluminescence. Figure S2: Correlation between parasite load by qPCR and lesion size. Figure S3: Simulated concentration‐time profiles. Figure S4: Dose‐stratified visual predictive checks. Figure S5: VPC parasite bioluminescence. Figure S6: VPC lesion size reduction. Figure S7: Goodness‐of‐fit plasma PK model. Figure S8: Goodness‐of‐fit tissue‐to‐plasma ratios. Figure S9: Goodness‐of‐fit parasite bioluminescence. Figure S10: Goodness‐of‐fit lesion size model. Figure S11: Model‐predicted parasite reduction. Figure S12: Model‐predicted lesion size reduction. Figure S13: Probability of target attainment for L. major parasite load reduction in humans following bid treatment for 7 or 10 days. Table S1: PTA results. [file CTS-19-e70535-s001.docx]

Supplementary materials

### Supplementary methods S1.

Thirty-six female BALB/c mice were infected with 4 × 10⁷ stationary-phase *L. major* Friedlin REH promastigotes (0.2 mL, subcutaneous injection) in the rump. Once the mice developed an average lesion diameter of 6.12 ± 0.90 mm and a bioluminescence signal of 1.35 × 10⁸ ± 7.64 × 10⁷, they were assigned to groups with similar bioluminescence signals (n=6). DNDI-6148 arginine monohydrate was administered via oral gavage at four dosage levels (6.25, 12.5, 25, and 50 mg/kg in 0.5% methyl cellulose (4000 cps), 0.1% sodium lauryl sulphate in Milli-Q water (pH 8), expressed as free base) for 10 consecutive days. A fifth group received vehicle only and served as the untreated control. A sixth group received 50 mg/kg paromomycin in PBS once daily. Plasma samples were collected from the tail vein on days 1 and 10 at 0.5, 1, 2, 4, 8, and 16 hours post-dose (n=3 per time point). At the last sampling time, the mice were sacrificed, and both infected and non-infected skin samples were excised. DNDI-6148 concentrations in plasma and tissue were quantified using a validated UPLC-MS/MS assay, with linear quantification ranges of 5.00–2000 ng/mL and 2.00–1000 ng/mL in human K2EDTA plasma and enzyme-digested tissue homogenate, respectively (6). Quantitative measurements of the bioluminescent signal emitted by transgenic Leishmania parasites within the mouse skin were obtained using the IVIS imaging chamber (IVIS Lumina XRMS system, Perkin Elmer). Briefly, 100 µL of d-luciferin (30 mg/mL in sterile water) was administered subcutaneously above the hind leg. Five minutes after injection, the mice were anesthetized with 2.5% isoflurane, and an additional five minutes later, they were placed in the imaging chamber where the bioluminescent signal was measured using exposure times ranging from 1 second to 5 minutes, depending on signal intensity. The Living Image software (Perkin Elmer) was used to define circular regions of interest to determine signal intensity. Each day's median blank bioluminescence signal measurement was subtracted from the corresponding parasite bioluminescence signal measurements to remove background measurement noise. Additionally, lesion size was measured daily in two perpendicular directions using digital callipers, and the average diameter was recorded. Lesion diameters were converted into lesion areas (mm²) using the equation for the area of an ellipse.

### Figure S1. Correlation between parasite load by qPCR and bioluminescence

End-of-treatment correlation between parasite load quantified by qPCR (expressed as parasites per mg of lesion tissue) and parasite bioluminescence signal. Skin lesion homogenates were prepared in 1 mL phosphate-buffered saline, of which 50 µL was processed by qPCR. Parasite counts were normalized to lesion weight (mg). For samples below the qPCR lower limit of quantification (LLOQ; 1,000 parasites per 50 µL), values were imputed as LLOQ/2 before normalization. Pearson’s correlation coefficient (R) and the linear regression equation are shown. The black solid line represents the fitted regression; the grey shaded area indicates the 95% confidence interval. Abbreviations: qPCR, quantitative polymerase chain reaction; p/s, photons per second.


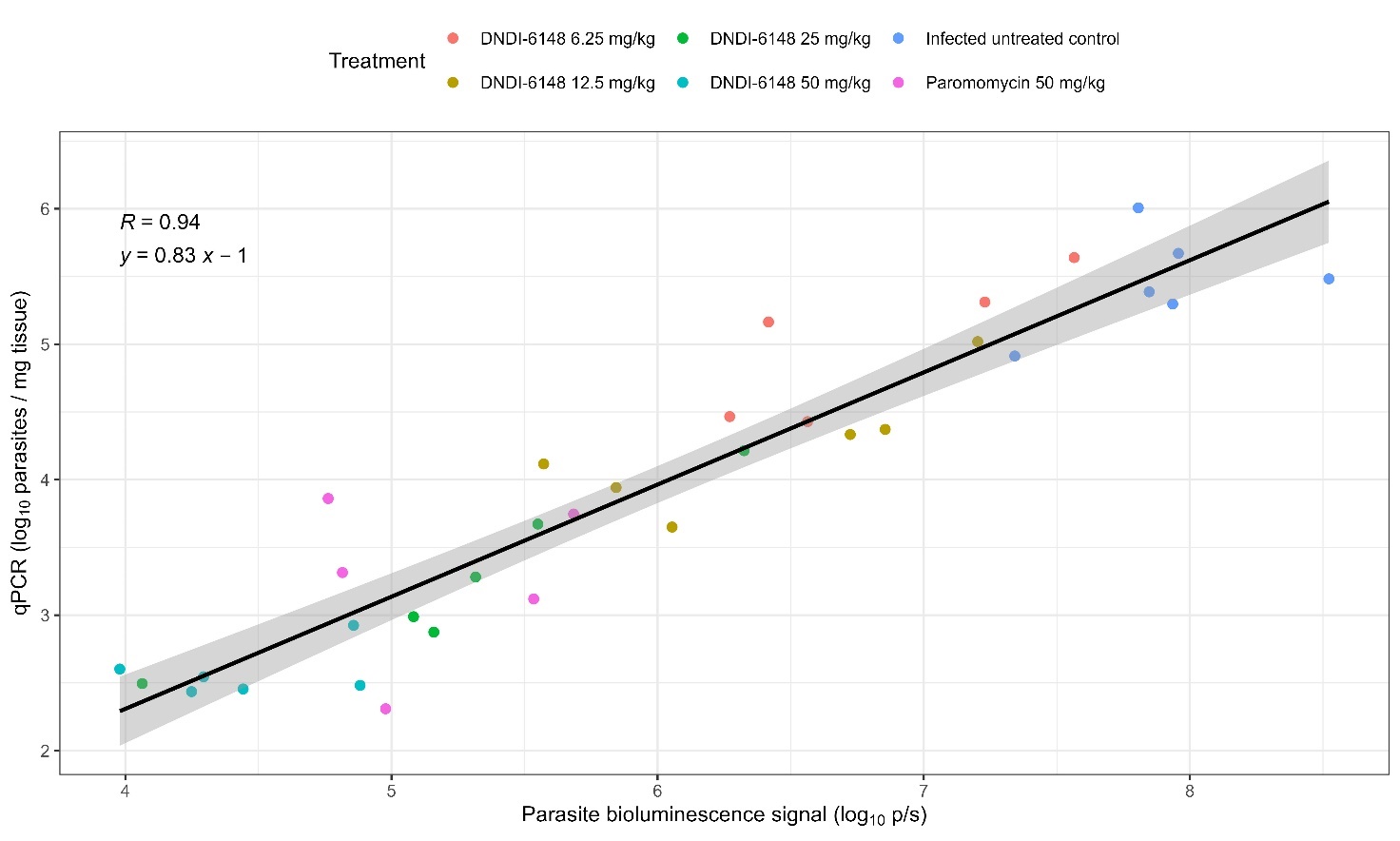


### Figure S2. Correlation between parasite load by qPCR and lesion size

End-of-treatment correlation between parasite load quantified by qPCR (expressed as parasites per mg of lesion tissue) and lesion size. Skin lesion homogenates were prepared in 1 mL phosphate-buffered saline (PBS), of which 50 µL was processed by qPCR. Parasite counts were normalized to lesion weight (mg). For samples below the qPCR lower limit of quantification (LLOQ; 1,000 parasites per 50 µL), values were imputed as LLOQ/2 before normalization. The vertical dotted line indicates the lower limit of quantification for lesion size (0.79 mm^2^). Pearson’s correlation coefficient (R) and the linear regression equation are shown. The black solid line represents the fitted regression; the grey shaded area indicates the 95% confidence interval. Abbreviation: qPCR, quantitative polymerase chain reaction.


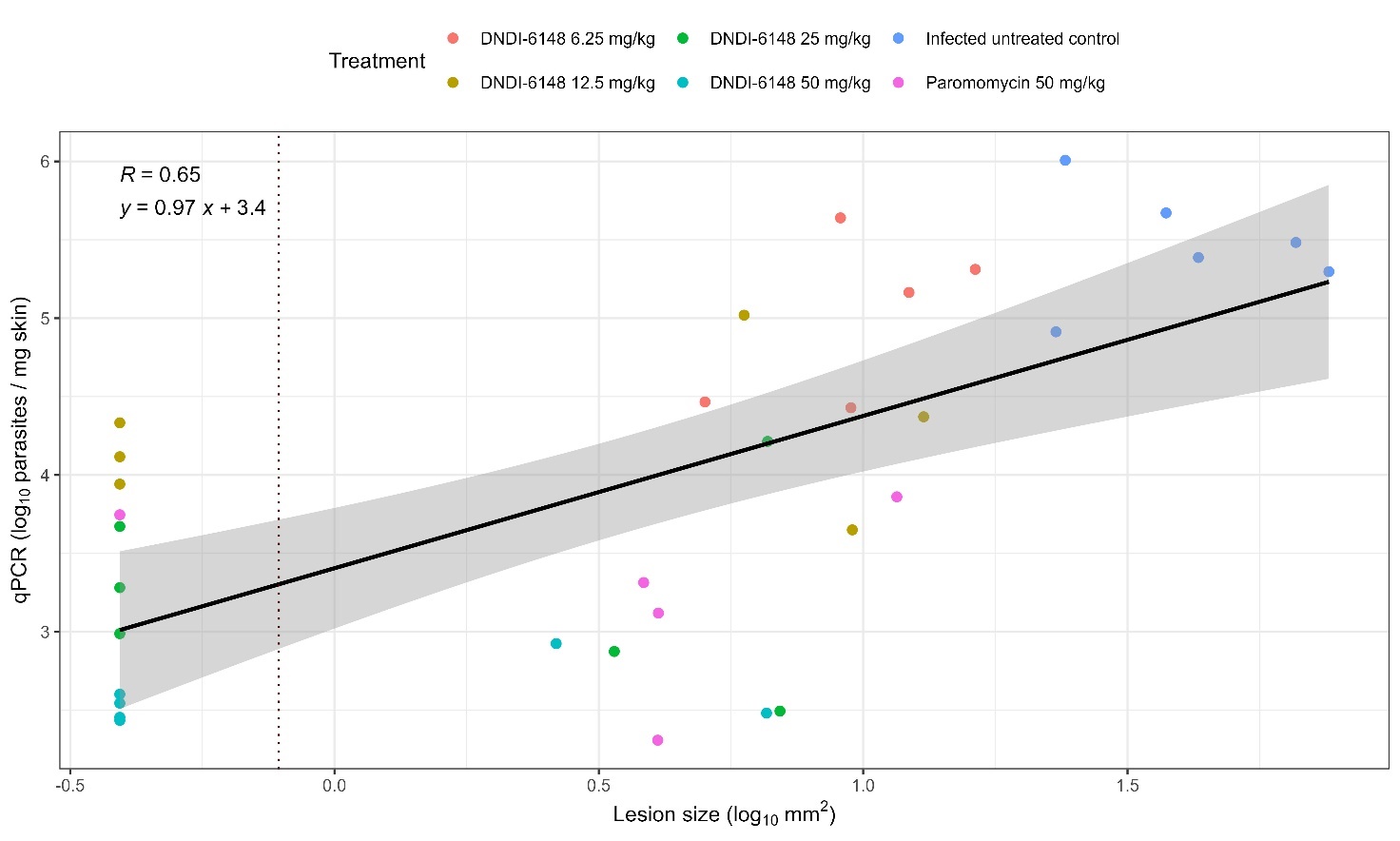


### Figure S3. Simulated concentration-time profiles

Simulated concentration-time profiles of a typical mouse receiving the studied dosing regimens (6.25–50 mg/kg, twice daily for 10 days), compared to the estimated *L. major* susceptibility (EC_50_). The x-axis represents time in days, while the y-axis shows the predicted free skin concentration of DNDI-6148 in µg/L. Solid lines represent the concentration-time profiles for each dosing regimen, with colours corresponding to different dose levels: red (6.25 mg/kg), yellow-green (12.5 mg/kg), blue (25 mg/kg), and purple (50 mg/kg). The dashed black line indicates the model-estimated EC_50_​ value (165 µg/L).


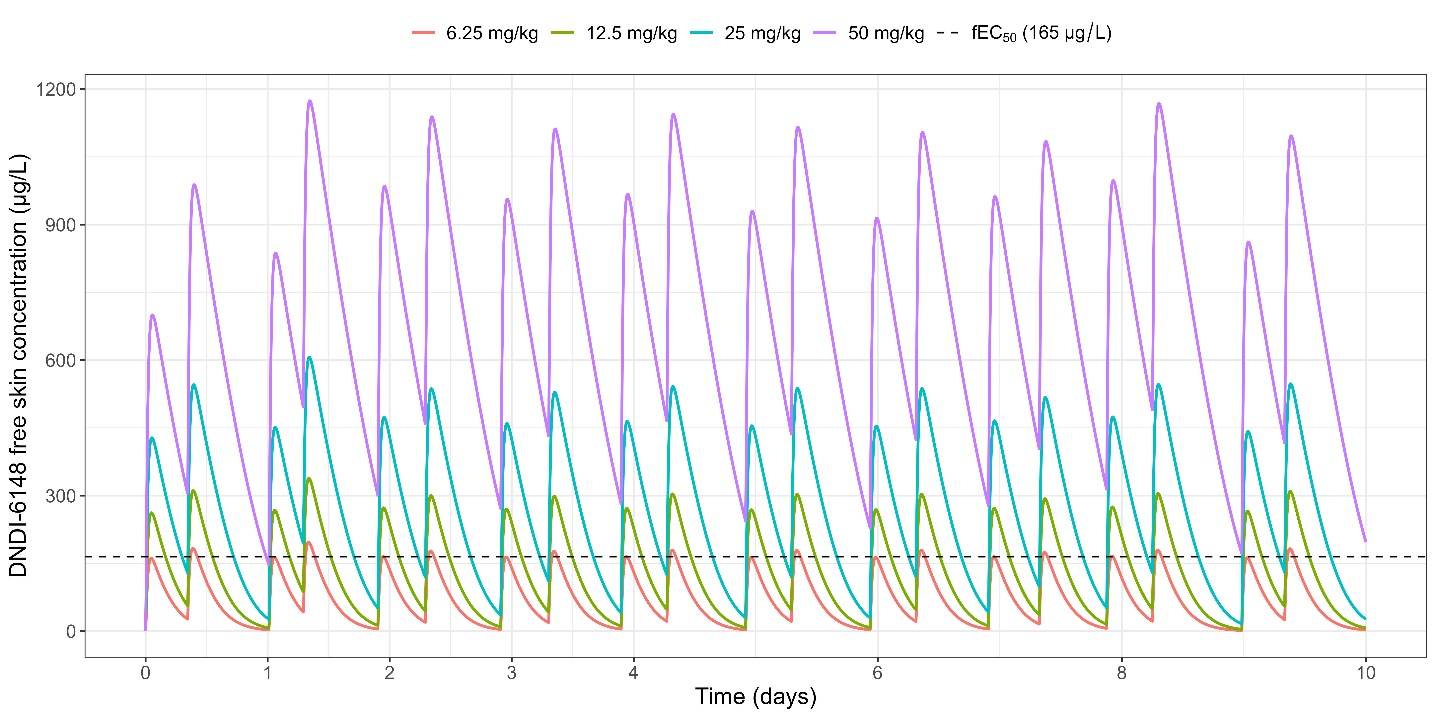


### Figure S4. Dose-stratified visual predictive checks

Dose-stratified visual predictive checks of the final DNDI-6148 plasma pharmacokinetic model, based on 1000 simulations. Black solid and dashed lines represent the median and 95% interval of the prediction-corrected observations; red shaded area represents the 95% CI of the median prediction; and blue shaded area represents the 95% CI of the 5th and 95th prediction intervals. CI: confidence interval.


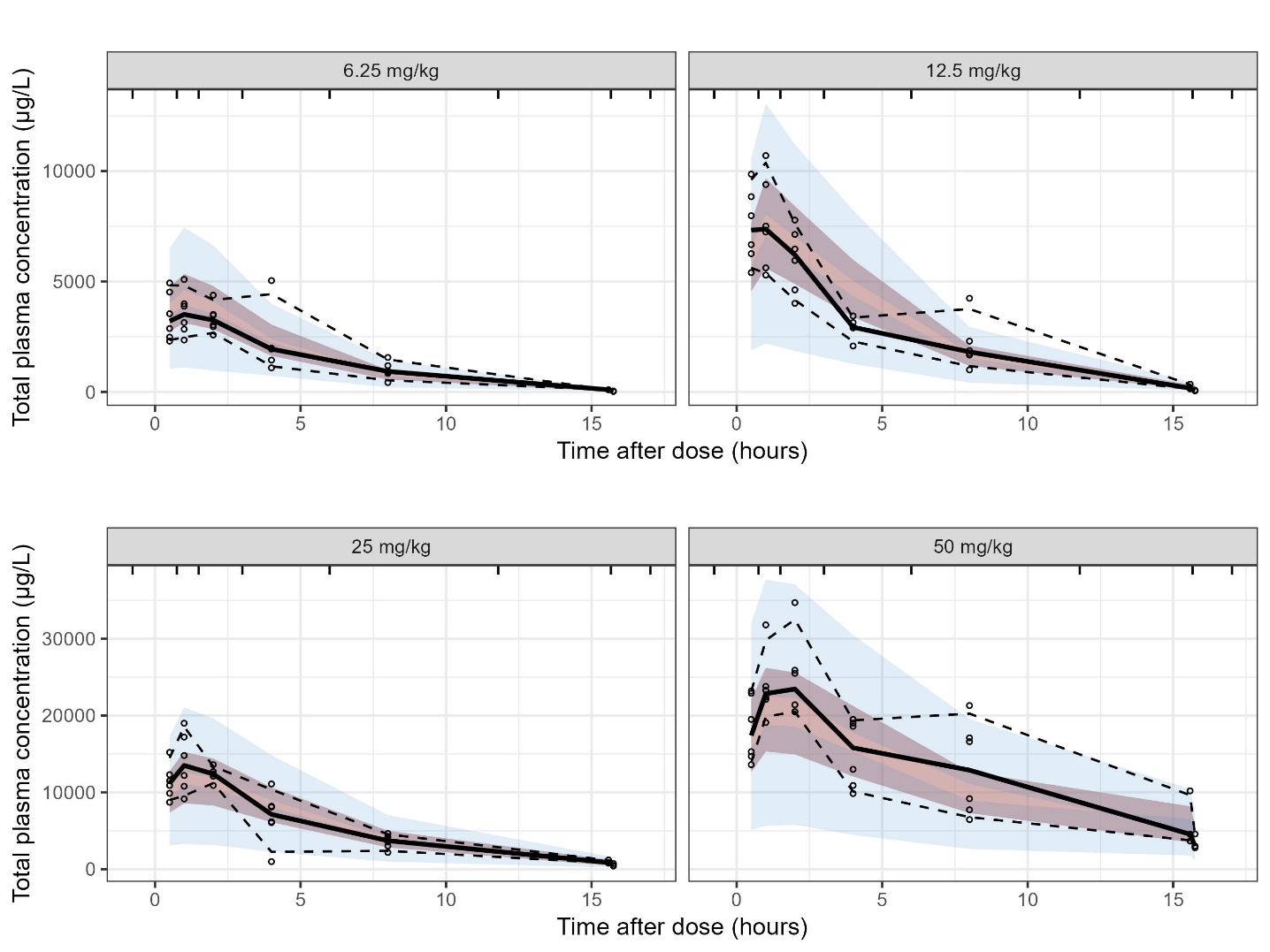


### Figure S5. VPC parasite bioluminescence

Dose-stratified visual predictive checks of the final DNDI-6148 parasite model, based on 1000 simulations. Black solid and dashed lines represent the median and 95% interval of the prediction-corrected observations; red shaded area represents the 95% CI of the median prediction; and blue shaded area represents the 95% CI of the 5th and 95th prediction intervals. Abbreviations: CI, confidence interval.


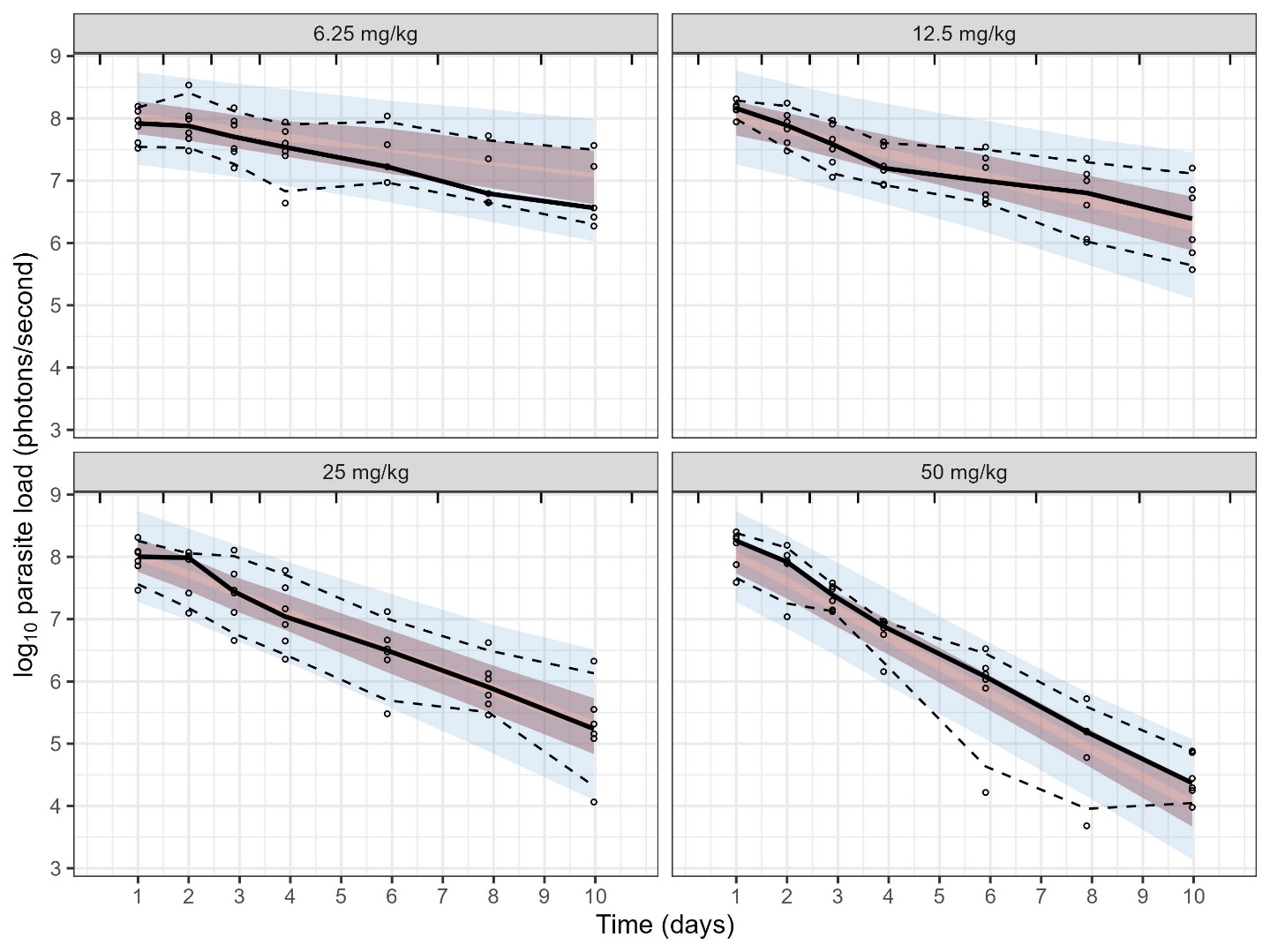


### Figure S6. VPC lesion size reduction

Dose-stratified visual predictive checks of the final DNDI-6148 lesion size model, based on 1000 simulations. Black solid and dashed lines represent the median and 95% interval of the prediction-corrected observations; red shaded area represents the 95% CI of the median prediction; and blue shaded area represents the 95% CI of the 5th and 95th prediction intervals. Abbreviations: CI, confidence interval.


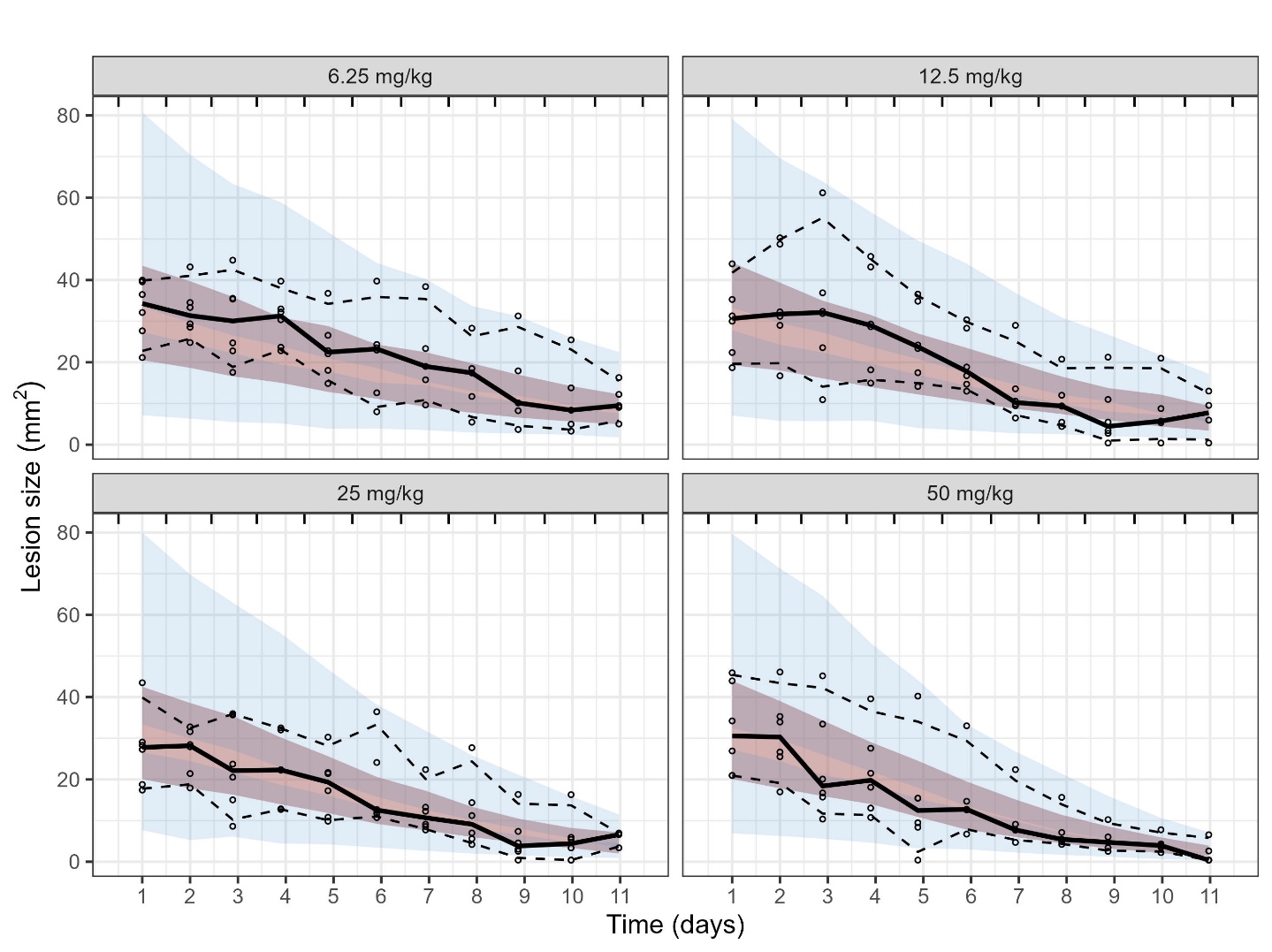


### Figure S7. Goodness-of-fit plasma PK model

Goodness-of-fit plots for the final DNDI-6148 total plasma pharmacokinetic model. (a) Observed versus population individually predicted DNDI-6148 plasma concentrations, (b) observed versus population predicted DNDI-6148 plasma concentrations, (c) conditional weighted residuals (CWRES) versus time after last dose, and (d) CWRES versus population predicted DNDI-6148 plasma concentrations.


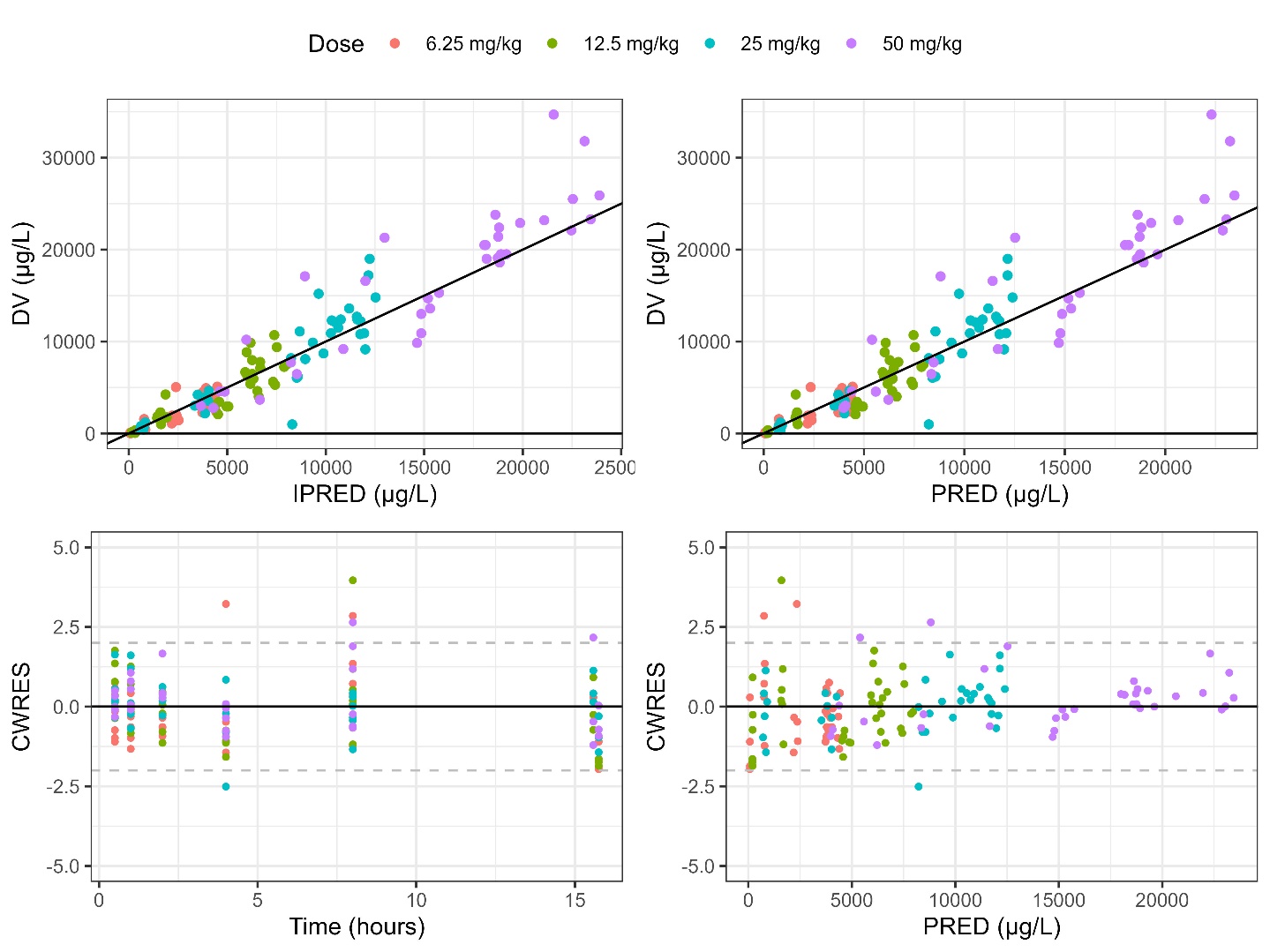


### Figure S8. Goodness-of-fit tissue-to-plasma ratios

Comparison of predicted and observed tissue-to-plasma ratios (R_tissue−plasma_​) for infected skin, non-infected skin, liver, and spleen. The x-axis represents predicted R_tissue−plasma_​, while the y-axis represents observed R_tissue−plasma_​, both presented on a logarithmic scale. Data points represent individual measurements, with tissues distinguished by colour and shape: infected skin (purple circles), non-infected skin (blue triangles), liver (brown squares), and spleen (green diamonds). The solid black identity line (y = x) denotes the line of unity, indicating perfect agreement between predicted and observed values.


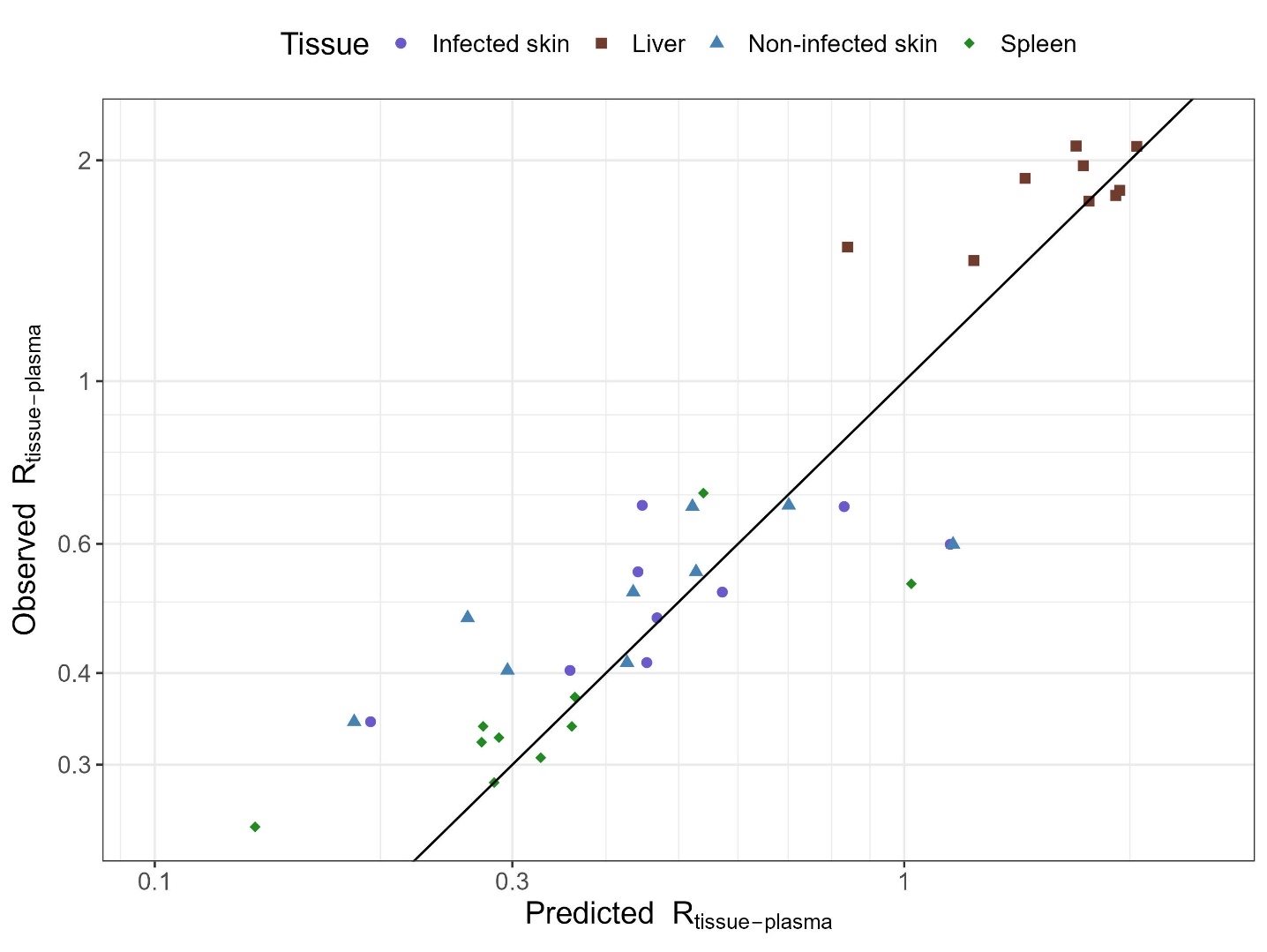


### Figure S9. Goodness-of-fit parasite bioluminescence

Goodness-of-fit plots for the final DNDI-6148 parasite bioluminescence model. (a) Observed versus population individually predicted parasite bioluminescence, (b) observed versus population predicted parasite bioluminescence, (c) conditional weighted residuals (CWRES) versus time after last dose, and (d) CWRES versus population predicted parasite bioluminescence.


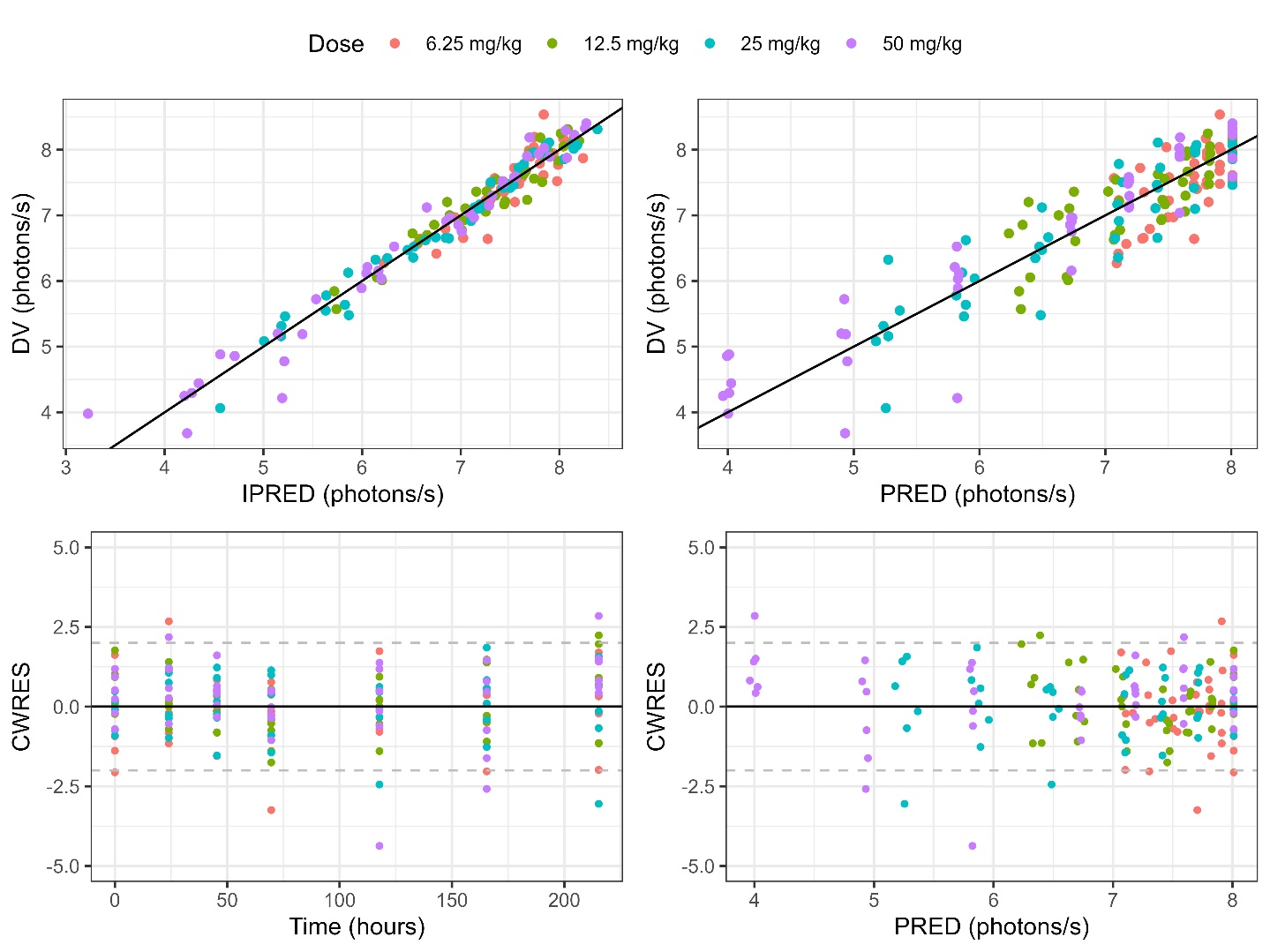


### Figure S10. Goodness-of-fit lesion size model

Goodness-of-fit plots for the final DNDI-6148 lesion size model. (a) Observed versus population individually predicted lesion size, (b) observed versus population predicted lesion size, (c) conditional weighted residuals (CWRES) versus time after last dose, and (d) CWRES versus population predicted lesion size.


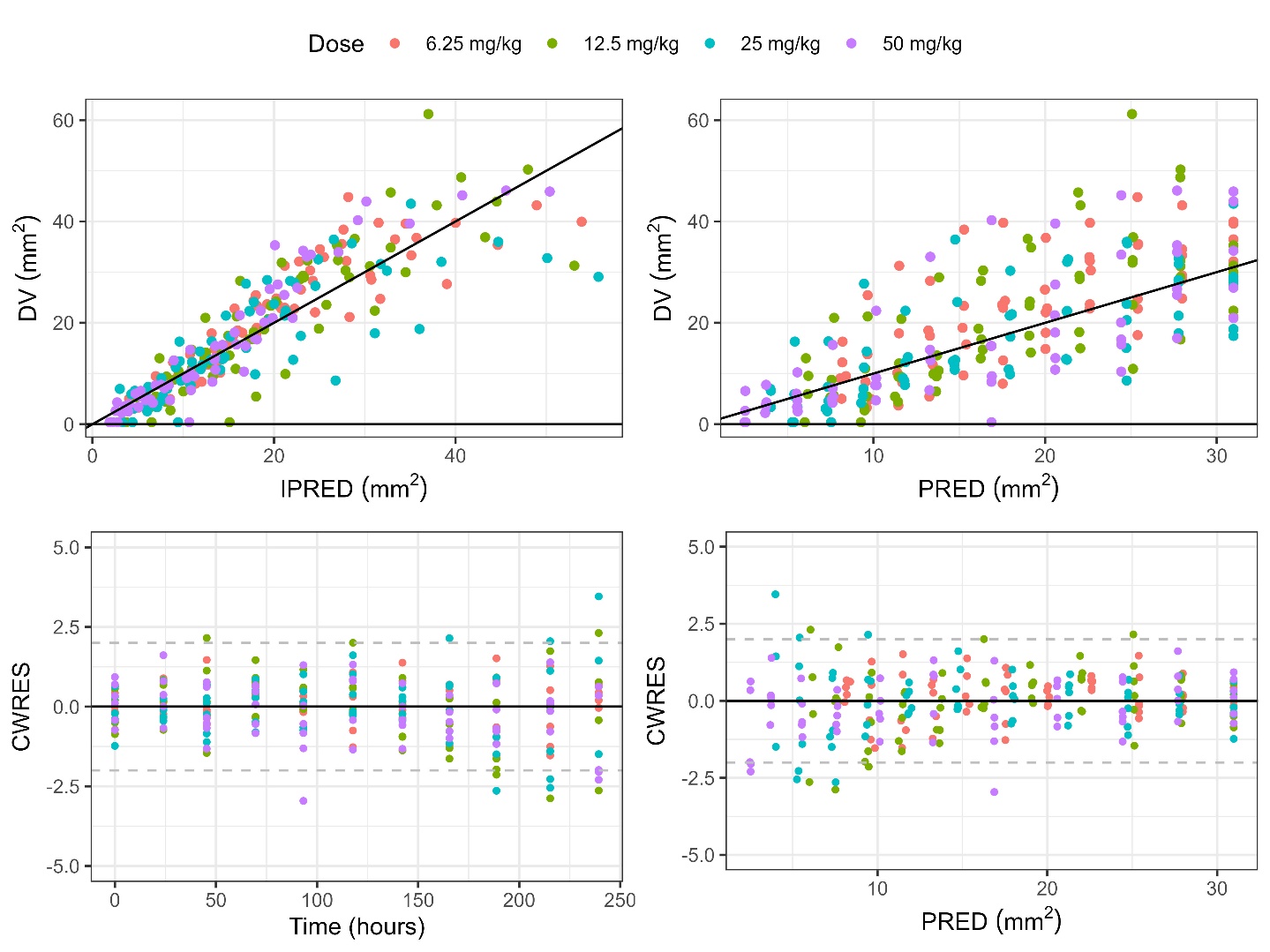


### Figure S11. Model-predicted parasite reduction

Simulated (n = 1000) percentage reduction in parasite load from baseline, incorporating between-subject variability, at the studied dose levels: 6.25 mg/kg (red curve), 12.5 mg/kg (blue curve), 25 mg/kg (green curve), and 50 mg/kg (purple curve), BID, 10-day treatment. The shaded area surrounding each curve represents the 50% confidence interval. Dotted and dashed lines indicate drug target profiles corresponding to 95% and 99% parasite reduction, respectively. Abbreviations: CI, confidence interval; BSV, between-subject variability.


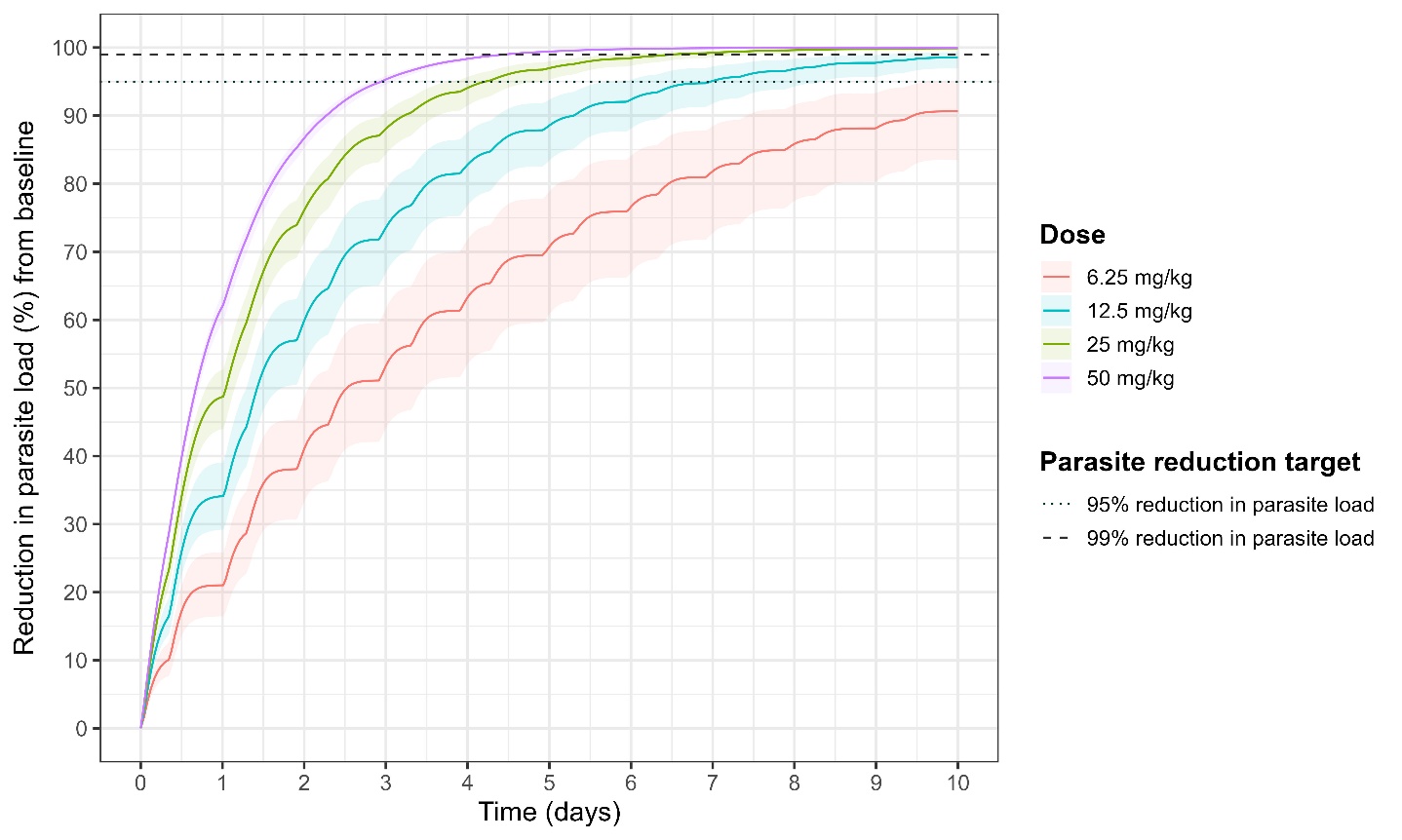


### Figure S12. Model-predicted lesion size reduction

Simulated (n = 1000) reduction in lesion size from baseline, incorporating between-subject variability (BSV), across the studied dose levels: 6.25 mg/kg (red curve), 12.5 mg/kg (blue curve), 25 mg/kg (green curve), and 50 mg/kg (purple curve), twice daily (BID), 10-day treatment. Shaded areas around each curve represent the 50% confidence interval (CI). Dashed line indicates the drug target profile of no visible lesion, defined as the lower limit of quantification (LLOQ). Abbreviations: CI, confidence interval; BSV, between-subject variability; LLOQ, lower limit of quantification.


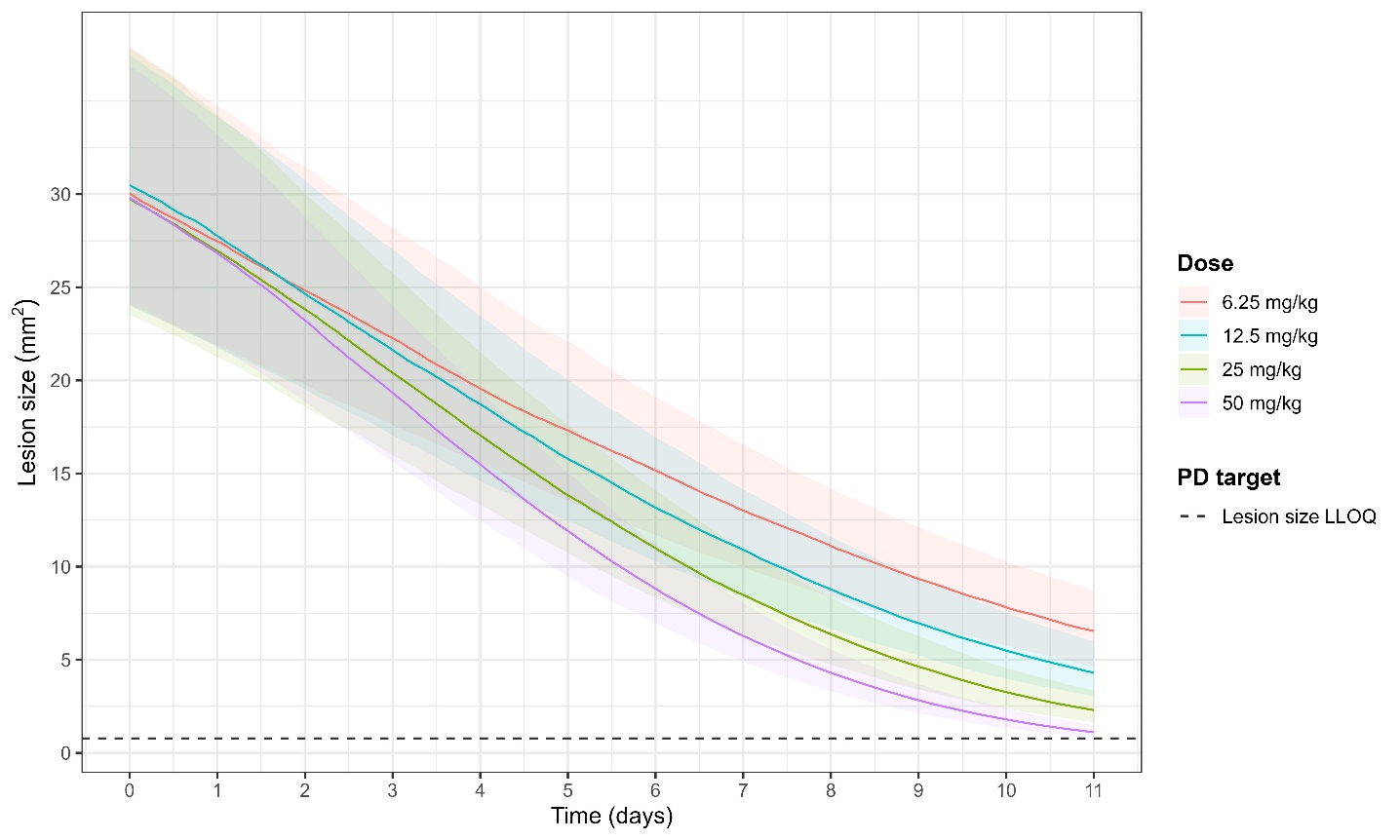


### Figure S13. Probability of target attainment for *L. major* parasite load reduction in humans following bid treatment for 7 or 10 days.

Model-predicted PTA for *L. major* parasite load reduction in humans following bid DNDI-6148 treatment for 7 or 10 days at doses ranging from 1.5 to 7 mg/kg (n = 10,000 per dose). The horizontal dashed line indicates that 90% of the simulated population has reached the specified target (95% or 99% parasite reduction from baseline), and the vertical dashed line represents the dose at which the model-predicted human AUC_0-24hr,ss_ exceeds that of the preclinical NOAEL. Abbreviations: PTA, probability of target attainment; bid, twice daily; d, days; NOAEL, no-observed adverse effect level.


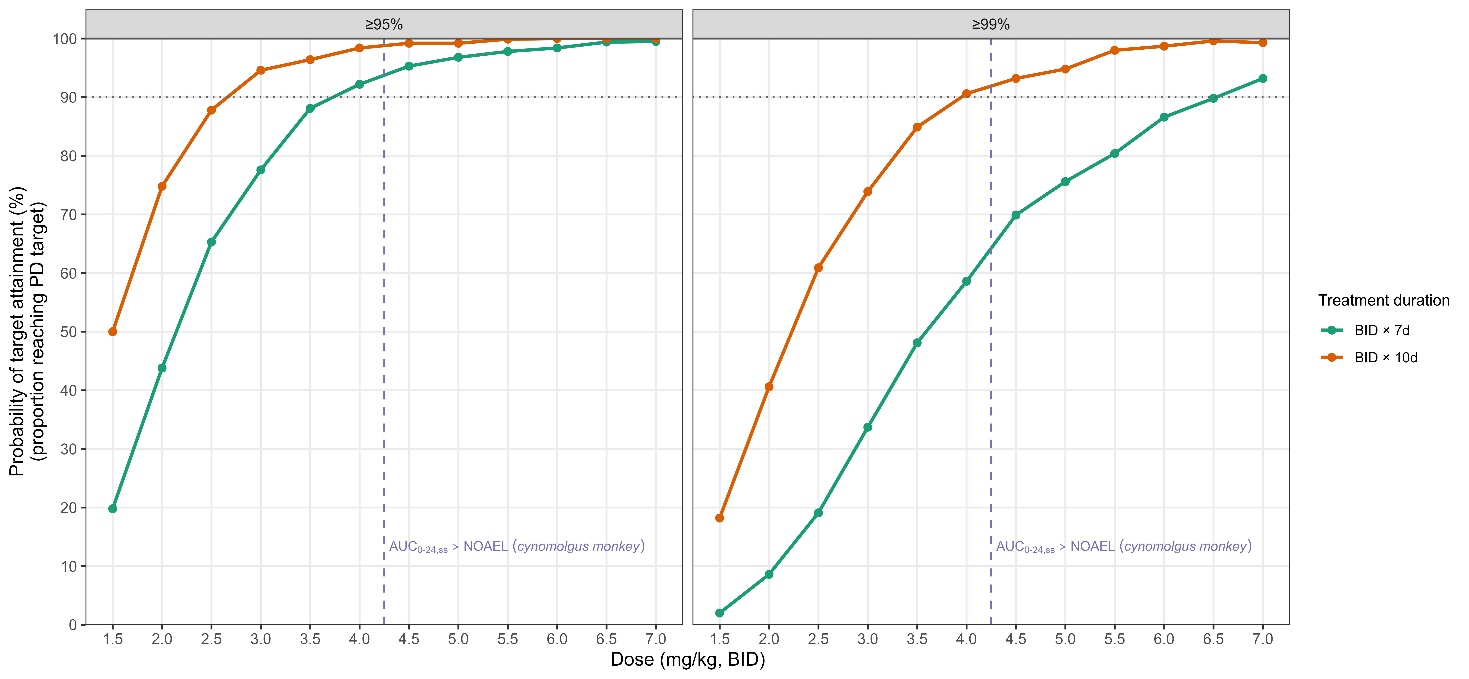


### Table S1. PTA results

Summary of the probability of pharmacodynamic target attainment (PTA) for different doses, treatment durations, and parasite reduction targets in a 70 kg patient. PTA values are highlighted in yellow and green if they exceed 90% and 95%, respectively. For each dosing scenario, 10000 individuals were simulated. Abbreviation: qd, once-daily administration.

| Dose | | Parasite reduction target from baseline | |
| --- | --- | --- | --- |
| Weight-based | Per 70 kg | 95% | 99% |
| 7 days treatment, qd | | | |
| 3.0 mg/kg | 210 mg | 23.4 | 3.50 |
| 4.0 mg/kg | 280 mg | 43.6 | 8.80 |
| 5.0 mg/kg | 350 mg | 67.7 | 23.6 |
| 6.0 mg/kg | 420 mg | 78.2 | 34.5 |
| 7.0 mg/kg | 490 mg | 86.5 | 49.0 |
| 8.0 mg/kg | 560 mg | 93.6 | 62.1 |
| 9.0 mg/kg | 630 mg | 95.0 | 70.0 |
| 10 mg/kg | 700 mg | 97.4 | 77.6 |
| 11 mg/kg | 770 mg | 98.6 | 82.5 |
| 12 mg/kg | 840 mg | 98.9 | 88.5 |
| 13 mg/kg | 910 mg | 99.2 | 90.5 |
| 14 mg/kg | 980 mg | 99.3 | 91.9 |
| 16 mg/kg | 1120 mg | 99.8 | 95.6 |
| 18 mg/kg | 1260 mg | 100 | 96.7 |
| 20 mg/kg | 1400 mg | 100 | 98.6 |
| 10 days treatment, qd | | | |
| 3.0 mg/kg | 210 mg | 55.0 | 20.0 |
| 4.0 mg/kg | 280 mg | 76.2 | 43.2 |
| 5.0 mg/kg | 350 mg | 89.4 | 63.3 |
| 6.0 mg/kg | 420 mg | 94.3 | 75.5 |
| 7.0 mg/kg | 490 mg | 97.6 | 85.7 |
| 8.0 mg/kg | 560 mg | 99.0 | 89.6 |
| 9.0 mg/kg | 630 mg | 99.2 | 94.6 |
| 10 mg/kg | 700 mg | 99.6 | 96.2 |
| 11 mg/kg | 770 mg | 99.8 | 97.3 |
| 12 mg/kg | 840 mg | 100 | 98.0 |
| 13 mg/kg | 910 mg | 100 | 98.7 |
| 14 mg/kg | 980 mg | 100 | 99.4 |
| 14 days treatment, qd | | | |
| 3.0 mg/kg | 210 mg | 76.6 | 48.5 |
| 4.0 mg/kg | 280 mg | 91.5 | 72.0 |
| 5.0 mg/kg | 350 mg | 96.6 | 85.7 |
| 6.0 mg/kg | 420 mg | 98.8 | 92.9 |
| 7.0 mg/kg | 490 mg | 99.5 | 96.4 |
| 8.0 mg/kg | 560 mg | 99.8 | 98.1 |
| 9.0 mg/kg | 630 mg | 99.9 | 98.9 |
| 10 mg/kg | 700 mg | 100 | 99.5 |
| 11 mg/kg | 770 mg | 100 | 99.8 |
| 12 mg/kg | 840 mg | 100 | 99.8 |
| 13 mg/kg | 910 mg | 100 | 99.9 |
| 14 mg/kg | 980 mg | 100 | 100 |
| 7 days treatment, bid | | | |
| 1.5 mg/kg | 105 mg | 19.8 | 2.00 |
| 2.0 mg/kg | 140 mg | 43.8 | 8.60 |
| 2.5 mg/kg | 175 mg | 65.3 | 19.1 |
| 3.0 mg/kg | 210 mg | 77.6 | 33.7 |
| 3.5 mg/kg | 245 mg | 88.1 | 48.1 |
| 4.0 mg/kg | 280 mg | 92.2 | 58.6 |
| 4.5 mg/kg | 315 mg | 95.3 | 69.9 |
| 5.0 mg/kg | 350 mg | 96.8 | 75.6 |
| 5.5 mg/kg | 385 mg | 97.8 | 80.4 |
| 6.0 mg/kg | 420 mg | 98.4 | 86.6 |
| 6.5 mg/kg | 455 mg | 99.4 | 89.8 |
| 7.0 mg/kg | 490 mg | 99.5 | 93.2 |
| 10 days treatment, bid | | | |
| 1.5 mg/kg | 105 mg | 50.0 | 18.2 |
| 2.0 mg/kg | 140 mg | 74.8 | 40.6 |
| 2.5 mg/kg | 175 mg | 87.8 | 60.0 |
| 3.0 mg/kg | 210 mg | 94.6 | 73.9 |
| 3.5 mg/kg | 245 mg | 96.4 | 84.9 |
| 4.0 mg/kg | 280 mg | 98.4 | 90.6 |
| 4.5 mg/kg | 315 mg | 99.2 | 93.2 |
| 5.0 mg/kg | 350 mg | 99.2 | 94.8 |
| 5.5 mg/kg | 385 mg | 99.9 | 98.0 |
| 6.0 mg/kg | 420 mg | 100 | 98.7 |
| 6.5 mg/kg | 455 mg | 100 | 99.6 |
| 7.0 mg/kg | 490 mg | 100 | 99.3 |
